# Multiplex CRISPR/Cas9 gene-editing platform in oil palm targeting mutations in *EgFAD2* and *EgPAT* genes

**DOI:** 10.1186/s43141-022-00459-5

**Published:** 2023-01-11

**Authors:** Bohari Bahariah, Mat Yunus Abdul Masani, Md Piji Mohd Al Akmarul Fizree, Omar Abd Rasid, Ghulam Kadir Ahmad Parveez

**Affiliations:** Advanced Biotechnology and Breeding Centre (ABBC), Malaysian Palm Oil Board (MPOB), No. 6, Persiaran Institusi, Bandar Baru Bangi, 43000 Kajang, Selangor Malaysia

**Keywords:** CRISPR/Cas9, Genome editing, Oil palm, *EgPAT*, *EgFAD2*

## Abstract

**Background:**

CRISPR/Cas9 is the most powerful and versatile genome-editing tool that permits multiplexed-targeted gene modifications for the genetic enhancement of oil palm. Multiplex genome-editing has recently been developed for modifying multiple loci in a gene or multiple genes in a genome with high precision. This study focuses on the development of high-oleic oil palm, the primary target trait for healthy low-saturated oil. To achieve this, the *fatty acid desaturase 2* (*FAD2*) and *palmitoyl-acyl carrier protein thioesterase* (*PAT*) genes, both of which are associated with fatty acid metabolism biosynthesis pathways in oil palm, need to be knocked out. The knockout of *FAD2* and *PAT* leads to an accumulation of oleic acid content in oil palms.

**Results:**

A total of four single-guide RNAs (sgRNAs) were designed in silico based on the genomic sequences of *EgFAD2* and *EgPAT.* Using robust plant CRISPR/Cas9 vector technology, multiple sgRNA expression cassettes were efficiently constructed into a single-binary CRISPR/Cas9 vector to edit the *EgFAD2* and *EgPAT* genes. Each of the constructed transformation vectors was then delivered into oil palm embryogenic calli using the biolistic, *Agrobacterium*-mediated, and PEG-mediated protoplast transformation methods. Sequence analysis of PCR products from 15 samples confirmed that mutations were introduced at four target sites of the oil palm *EgFAD2* and *EgPAT* genes. Single- and double-knockout mutants of both genes were generated, with large and small deletions within the targeted regions. Mutations found at EgFAD2 and EgPAT target sites indicate that the Cas9/sgRNA genome-editing system effectively knocked out both genes in oil palm.

**Conclusion:**

This technology is the first in oil palm to use CRISPR/Cas9 genome-editing to target high-oleic-associated genes. These findings showed that multiplex genome-editing in oil palm could be achieved using multiple sgRNAs. Targeted mutations detected establish that the CRISPR/Cas9 technology offers a great potential for oil palm.

**Supplementary Information:**

The online version contains supplementary material available at 10.1186/s43141-022-00459-5.

## Background

Oil palm (*Elaeis guineensis* Jacq.) is a very productive crop. It produces high-quality oil that is rich in natural chemical compounds that are essential for health and nutrition. In 2020, oil palm was the world’s most consumed edible oil. It contributes approximately 34.3% of the world’s production of oils and fats, followed by soybean and rapeseed oil [1]. Global oil and fat production are increasing year after year, paralleling the rising demand from the world’s growing population. In response to the demands and growth trend, oil palm yield improvement is essential. The adoption of breeding and genomic technology to increase yield and productivity is required to minimize land expansion. Palm oil comprises 50% saturated fatty acids, consisting of 44% palmitic acid, 5% stearic acid, and trace amounts of myristic acid. Palm oil also contains 40% monounsaturated oleic acid and 10% polyunsaturated linoleic and linolenic acids [2].

Higher levels of oleic acid in palm oil are often desirable for industrial applications and have a great deal of importance in human nutrition. A high level of monounsaturated fat in high-oleic acid oil was shown to reduce low-density lipoprotein cholesterol without lowering high-density lipoprotein cholesterol. This helps prevent lifestyle diseases such as heart attacks and strokes [3–5]. In addition, high oleic acid would enhance the physical properties, improving shelf stability and nutritional value. Therefore, the high demand for premium-quality oils such as oleic acid among the health-conscious population may improve the odds of Malaysian palm oil penetration into developed countries.

Conventional breeding has been used to enhance oleic acid content and reduce the levels of linoleic acid in palm oil [6, 7]. However, conventional breeding techniques are laborious and time-consuming. At least 20 years are required for the generation of trait-improved oil palms [8]. Therefore, conventional genetic engineering has been used to produce transgenic oil palms with high oleic acid contents [6, 7, 9]. This was done by silencing two key enzymes, oleoyl-CoA desaturase (EgFAD2) and palmitoyl-ACP-thioesterase (EgPAT), that regulate the composition of oil palm fatty acids [6, 7, 9]. However, the research efforts have yet to result in stable lines, and the development of transgenic oil palm is still highly challenging [10]. In oil palm, EgFAD2 is present in two copies, namely EgFAD2-1 and EgFAD2-2. The *EgFAD2-1* gene is located on chromosome 8 and is highly expressed at the later stages of mesocarp tissue development, 15, 17, and 20 weeks after anthesis (WAA), with the strongest level at 15 WAA. The expression was correlated with the levels of linoleic acid deposited in the mesocarp as the fatty acids began to accumulate in the mesocarp tissues at 15 WAA. EgFAD2 is important in oil storage because oil deposition begins in oil-bearing tissue at week 15 [2]. On the other hand, the *EgPAT* gene, also known as the *EgFATB* gene in oil palm, has been classified into four types, namely *EgFATB-1*,* EgFATB-2*, *EgFATB-3*, and *EgFATB-4*. The four variations of the *EgFATB* gene show differences in their expression patterns and functions in fatty acid biosynthesis. The *EgFATB-1* gene is highly expressed in 15WAA kernels and in 10WAA mesocarp tissues, whereas *EgFATB-2* is expressed mainly in the mesocarp and early stages of kernel development at 15WAA. While *EgFATB-4* is not expressed in either tissue, the *EgFATB-3* gene is expressed in all transcriptome libraries [11]. With the availability of the complete oil palm genome sequence data released in 2013 [12], genome-editing technology such as clustered regularly interspaced short palindromic repeats (CRISPR) and CRISPR-associated protein 9 (Cas9) has opened the possibility of targeted modification of the oil palm genome for future trait improvement. The adoption of CRISPR/Cas9 genome editing is highly needed to address the challenges and reassert the potential of genetic engineering for oil palm improvement. A recent study by Yeap et al. [13] has employed CRISPR/Cas9 to target two genes that affect the phenotype of oil palm: phytoene desaturase (*EgPDS*) and brassinosteroid-insensitive 1 (*EgBRI1*). Based on the observation of premature necrotic shoots and stunted phenotypes, this approach may be successfully used for oil palm genome editing.

A CRISPR/Cas9 system based on an adaptive defence system in bacteria performs robust genome modification using a single-guide RNA (sgRNA) and Cas9 nuclease [14]. The sgRNA directs the Cas9 nuclease to a specific genomic site to create double-stranded DNA breaks (DSBs). The plant’s endogenous repair system fixes the DSBs by non-homologous end joining, resulting in the insertion or deletion of nucleotides. The repair system also induced homologous recombination, leading to gene replacements and insertions [15]. The ability of CRISPR/Cas9, particularly using multiple sgRNAs, to edit multiple genes at different locations at once is vital due to the complexities of the polyploidy genome of oil palm. Several FAD2 knockout mutants have been generated using the multiplex CRISPR/Cas9 system, such as in pennycress (*Thlaspi arvense* L.) [16], rice (*Oryza sativa* L.) [17], tobacco (*Nicotiana tabacum* L.) [18], peanut (*Arachis hypogaea* L.) [19], soybean (*Glycine max* L.) [20, 21], rapeseed (*Brassica napus*) [22], and Camelina (*Camelina sativa* L.) [23, 24].

In this study, a multiplex CRISPR/Cas9 system platform was employed to induce mutations in the *EgFAD2* gene located on chromosome 8 and in the *EgPAT* gene, also known as the *EgFATB-1* gene, located on chromosomes 3 and 7. This approach is projected to limit the activity of the PAT and FAD2 enzymes, resulting in an elevation in oleic acid composition and a reduction in linoleic acid and other polyunsaturated fatty acids. Preliminary findings demonstrated that the multiplex CRISPR/Cas9 system successfully mutated both the *EgFAD2* and *EgPAT* genes. Several mutations were observed, including large and small deletions of the coding sequence in the targeted genes. To our knowledge, this is the first report of oil palm genome editing with evidence of mutations in the *EgFAD2* and *EgPAT* genes.

## Methods

### Selection of target sequences

The sequences of oil palm *Elaeis guineensis FAD2* (GenBank accession: KT023602) and *PAT* genes (GenBank accession: DQ422858) were downloaded from the Genbank (https://www.ncbi.nlm.nih.gov/genbank). Two sgRNAs for *EgFAD2* and two sgRNAs for *EgPAT* gene sequences were chosen by initially identifying the NGG protospacer adjacent motif (PAM) sequence, followed by 20 nucleotides upstream of the PAM sites [5’-20nt-NGG]. sgRNA with no more than four continuous Ts (TTTT) was designed using CRISPR-GE software (http://skl.scau.edu.cn/) based on the coding region sequence. In addition, potential target sequences with GC content between 30 and 65% were preferred due to higher editing efficiency (Table S1) [14, 25]. The sgRNA sequences were aligned and validated by using BLAST at the National Center for Biotechnology Information (NCBI) (https://blast.ncbi.nlm.nih.gov/Blast.cgi).

### Analysis of nucleotide composition and secondary structure

Secondary structure analysis of the candidate target-sgRNA sequences was carried out with the RNA Mfold version 2.3 webserver (http://mfold.rna.albany.edu/?q=mfold/RNA-Folding-Form2.3) to predict the RNA folding and interactions between the sgRNA and the scaffold region [26]. The sgRNA sequence, 20 nucleotides upstream from the PAM site, and the scaffold sequence (TTTAGAGCTAGAAATAGCAAGTTAAAATAAGGCTAGTCCGTTATCAACTTGAAAAAGTGGCACCGAGTCGGTGCTTTTTTT) were analyzed at a temperature of 37 °C. The number of base pairings of the guide sequence with target sequences in the secondary structures obtained was further investigated. The internal base pairs (IBPs), consecutive base pairs (CBPs), and total base pairs (TBPs), in the guide sequence, were calculated.

### *In vitro* transcription and screening of sgRNA

The sgRNAs designed for targeting the *EgFAD2* and *EgPAT* genes were evaluated based on target cleavage efficiency using the Guide-it™ sgRNA In Vitro Transcription kit and the Guide-it™ Screening Systems Kit (Takara Bio Inc.) according to the manufacturer’s instructions.

### PCR amplification of DNA templates for sgRNA synthesis

The 56- to 58-nt forward primers to create the DNA template were designed (Table S2) to contain a T7 promoter sequence plus four extra bases (5′-CCTCTAATACGACTCACTATA, 21 total nt) upstream of the primer. A transcription initiation site of at least two guanines (GG) in front of the target sequences is required. By using the Guide-it scaffold template and the appropriate forward primer in a PCR reaction, a DNA template containing the sgRNA-encoding sequence under the control of a T7 promoter was created. PCR was performed as follows: 33 cycles of 10 s at 98 °C, followed by 10 s at 68 °C. The amplified product was fractionated with 1% agarose gel electrophoresis.

### *In vitro* transcription

The in vitro transcription was carried out in a 20-μl reaction mixture consisting of 7 μl of Guide-it in vitro transcription buffer and 5 μl of PCR products (sgRNA PCR template) for 4 h at 37 °C, and 3 μl of T7 RNA polymerase. Sterile distilled water was added to obtain a 20 μl final volume per reaction. The transcription product was then purified following the manufacturer’s instructions. The concentration of the purified sgRNA was determined using a NanoDrop 2000.

### *In vitro* cleavage assay

A cleavage reaction was performed using purified sgRNA combined with the Guide-it recombinant Cas9 nuclease for 5 min at 37 °C. The Cas9/sgRNA complex created was mixed with 100–250 ng of PCR-amplified DNA fragment from oil palm using primers designed in Table S2 under the following conditions: 98 °C for 2 min, and then 35 cycles of 98 °C for 10 s, 60 °C for 15 s, and 68 °C for 1 min. The mixture was incubated for 1 h at 37 °C, followed by 5 min at 80 °C. The cleavage reaction was analyzed by agarose gel electrophoresis.

### Multiplex CRISPR Vector Assembly

PCR amplification for assembling sgRNA expression cassettes was carried out using PrimeSTAR GXL (Clontech Laboratories, USA). Ligation of sgRNA expression cassettes into the CRISPR/Cas9 vector was performed using the ClonExpress MultiS One Step Cloning Kit (Vazyme Biotech Co., Ltd.). The isolation of plasmids was conducted using Nucleospin Plasmid Easy Pure (Macherey Nagel) and analyzed by restriction enzyme digestion (New England Biolabs).

### sgRNA cloning

As indicated in Table S2, the target adaptor primer pairs in the sgRNA expression vectors (pYLsgRNA-OsU6a/b) were synthesised and annealed to generate double-stranded fragments with the required overhangs of the respective U6 promoter. The adaptor primers were diluted and mixed to a final concentration of 10 μM, denatured at 100 °C in boiling water for 1 min, and cooled to room temperature over 2 h. The target adaptors were ligated into the *Bsa*I digested pYLsgRNA-OsU6a or pYLsgRNA-OsU6b intermediate vector using the following protocol: a reaction mixture comprised of 1 μg pYLsgRNA-OsU6a/b digested with 10 U *Bsa*I (NEB), 1 μl of 10 × T4 DNA ligase buffer in the presence of 400 U T4 ligase, and 1 μl of annealed primer (10-μM stock) was incubated at room temperature (~ 24 °C) for 20 min. Then, the ligated products were used as templates to be assembled into the pYLCRISPR/Cas9 binary plasmid via Gibson assembly [27].

### Cloning of the Gibson Assembly

The ligated products were amplified by the first round of two PCRs using the primer sets U-F/sgRNA RP (PCR 1) and sgRNA FP/gR-R primer (PCR 2). U-F and gR-R are the universal primers, while FP and RP are the forward and reverse primer adaptors (Table S2). The reaction was performed in a 15-μl mixture containing 3 μl of 5 × PrimeSTAR GXL buffer, 1.5 μl of 2.5 mM dNTP mix, 0.25 μl of 10 μM primers, 0.5 μl of a template (10 ng/μl), and 0.4 μl of PrimeSTAR GXL DNA polymerase (5 U/μl). Distilled water was added to obtain a total volume of 15 μl. The PCR used a standard 3-step PCR protocol with an initial denaturation step of 30 s at 98 °C, 30 cycles of 10 s at 98 °C, 15 s at 60 °C, and 20 s at 68 °C, and 1 cycle at 68 °C at 2 min. The products from the first round of PCRs were then used as templates in the second round of PCR, using the nested position-specific primers (U-F and gR-R). The nested PCR products were then amplified using site-specific primer pairs (U-GA and Pgs-GA) (Table S2) to obtain a complete sgRNA expression cassette. The primer pairs depend on sgRNA expression cassettes to be amplified. In this study, four primer pairs (U-GAL/Pgs-GA2, U-GA2/Pgs-GA3, U-GA3/Pgs-GA4, and U-GA4/Pgs-GAR) were used to amplify four sgRNA expression cassettes (U6aT2Fad2-U6aT2PAT-U6bT2Fad2-U6bT2PAT). The PCR-amplified fragment and the pYLCRISPRCas9 digested vector were incubated for 45 min at 37 °C with Exnase II (Vazyme Biotech Co., Ltd.). Twenty microliters of the resulting recombination reaction was used to transform DH5α competent cells using a standard transformation procedure. Positive clones were picked and analyzed by *Asc*I digestion to confirm the successful insertion of the target sequence into the sgRNA expression cassette. Using SP-L and SP-R primers (Table S2), the clones with validated product lengths were sequenced.

### Oil palm transformation and growth conditions

The CRISPR/Cas9 constructs were introduced into oil palm calli using biolistic and *Agrobacterium*-mediated transformation, while PEG-mediated transfection was used to introduce the constructs into oil palm protoplasts according to the established protocol [6, 28–30]. The calli were then selected for regeneration on 4 mg liter^−1^ of hygromycin selective agent under 16/8 h light conditions at 28 °C. The fluorescence stereomicroscope Multizoom AZ-100 (Nikon, Japan) with an ET-mCherry, Texas Red® filter set (Chroma, USA), was used to visualize the red fluorescent signals on oil palm calli co-bombarded with the pAMDsRED plasmid.

### Screening and confirmation of transgenic oil palm

The modified Edward method [31] was used to extract DNA from protoplasts and embryogenic calli of transformed oil palms, followed by amplification of targeted regions by PCR. The PCR products were directly used for sequencing using specific primers (Table S2), with binding positions around 150–250 bp upstream of the target sites. The DNA template was also used to determine the presence of transgenes, *Cas9*, and *H* genes using corresponding primers, as listed in Table S2. To confirm the transgenic status, the amplified PCR products were fractionated on a 1% agarose gel. Chromatograms from Sanger sequencing results generated from PCR products were analyzed using SnapGene (GSL Biotech LLC, USA) and the ICE software (Synthego, USA) (https://ice.synthego.com) [32]. By comparing and decomposing Sanger traces, the ICE Analysis was used to analyse the kind of mutation (indel) and editing efficiency (contribution) in the studied populations.

### RT-PCR assay

The total RNA was extracted from untransformed (wild type) and transformed oil palm embryogenic calli using the RNAEasy Plant Mini Kit (Qiagen, USA). The RNA template is converted into cDNA by the enzyme reverse transcriptase using the iScript cDNA Synthesis Kit (BIORAD). The cDNA later serves as a template for PCR amplification. The primers used in RT-PCR are provided in Table S2. RT-PCR cycles were programmed at 95 °C for 5 min, 30 cycles of 95 °C for 45 s, 57 °C for 45 s, 72 °C for 45 s, and 1 cycle of 72 °C for 5 min. The oil palm *actin* gene was used as the internal control to confirm the quality and integrity of the cDNAs. The RT-PCR products were evaluated by agarose gel electrophoresis.

## Results

### sgRNA selection and CRISPR/Cas9 vector construction

**Fig. 1:**
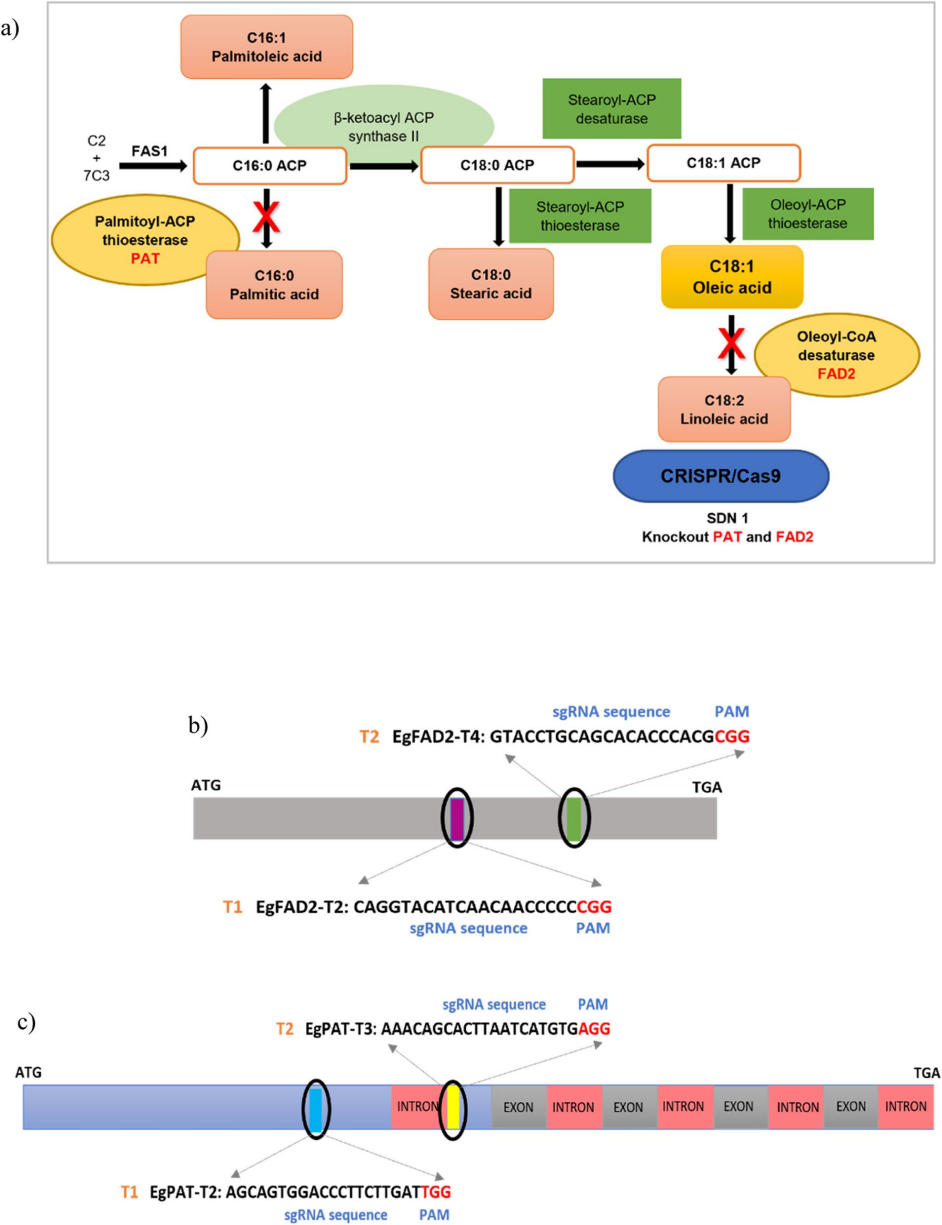
Schematic representation of CRISPR/Cas9-mediated targeted mutagenesis in the oil palm *EgFAD2* and *EgPAT* genes (**a**). The simplified metabolic pathway for the biosynthesis of fatty acids C16:0 and C18:0 in oil palm *PAT* and *FAD2* genes were knocked out (X) to prevent palmitate from being diverted to TAG and oleate from being desaturated into linoleic acid. The target genes’ sgRNA target sites are depicted in a schematic diagram. Schematic diagram of the targeting sequence (**b**) Two sgRNAs (T1 and T2) of the *EgFAD2* gene correspond to locations in the coding region, and (**c**) two sgRNAs (T1 and T2) of the *EgPAT* gene match to first and second exon locations, respectively. Introns are shown as pink boxes, and exons are shown as grey and blue boxes

This study has developed a CRISPR/Cas9 system to generate knockout mutations within *EgPAT* and *EgFAD2*, which are both involved in C16:0 and C18:0 biosynthesis, respectively (Fig. 1a). To extensively evaluate the efficiency of the CRISPR/Cas9 system in oil palm, multiple sgRNAs were selected to induce mutations in the *EgFAD2* and *EgPAT* genomic sequences. The four most efficient sgRNAs were designed, namely EgFAD2-T1, EgFAD2-T2, EgPAT-T1, and EgPAT-T2, which are adjacent to the PAM sequence of NGG, and were selected using the CRISPR-GE online tool (Fig. 1b, c; Table S1). The sgRNA sequences were also explored using BLAST to minimize the probability of off-target effects (data not shown). The GC content of the four target sites was chosen to be 35%, 55%, 60%, and 65% (Table S1). The secondary structure of all sgRNAs (Fig. S1) was evaluated by the Mfold web server prediction based on the minimum free energy (MFE) algorithm [33]. Secondary structure analysis found that all four guide sequences have 2–9 consecutive base pairs (CBP) and total base pairs (TBP) between the target sites with sgRNA backbones (Table S1). Recent research implies that secondary structures with more than 12 TBP and 7 CBP will limit editing efficiency, as seen in rice [32].

**Fig. 2:**
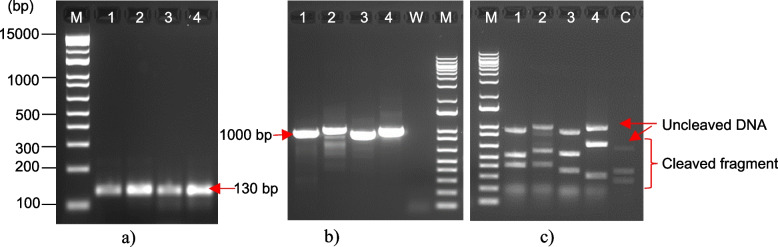
In vitro assay to evaluate cleavage efficiency by the Guide-it sgRNA screening kit. (**a**) PCR products from the amplification of *EgFAD2* (lane 1: sgRNA1, lane 2: sgRNA2) and *EgPAT* (lane 3: sgRNA3, lane 4: sgRNA4) sgRNA templates at 130 bp for in vitro transcription. (**b**) PCR products are amplified from oil palm genomic DNA containing sgRNA target sites; the PCR templates of candidate sgRNAs are then in vitro combined with a recombinant Cas9 for a fragment cleavage site (**c**). EgFAD2-T1 (lane 1), EgFAD2-T2 (lane 2), EgPAT-T1 (lane 3), and EgPAT-T2 (lane 4) represent the PCR fragments with 921 bp, 986 bp, 903 bp, and 978 bp length, respectively. Cleavage fragments were present for all sgRNAs; they were cleaved into two fragments by the sgRNA/Cas9 complex: 503 bp and 395 bp for EgFAD2-T1; 559 bp and 404 bp for EgFAD2-T2; 531 bp and 349 bp for EgPAT-T1; and 287 bp and 668 bp for EgPAT-T2. Lane C is a control fragment that has a size of 614 bp and cleaved DNA of 350 bp and 264 bp. Lane W is a negative control, and lane M is a 1 kb plus DNA marker (Invitrogen)

After designing sgRNAs targeting the *EgFAD2* and *EgPAT* genes, the sgRNAs were validated in vitro to assess their cleavage efficiency. First, DNA templates for transcription of the selected sgRNAs were prepared by PCR amplification. The transcription templates encoding the T7 promoter region were subsequently used to transcribe the DNA template into RNA. The in vitro transcribed sgRNAs were then incubated with Cas9 nuclease to form Cas9-sgRNA complexes. After that, a target DNA fragment was added, and the cleaved dsDNA was analyzed by gel electrophoresis. The results showed that the cleaved fragments were observed for EgFAD2-T1, EgFAD2-T2, EgPAT-T1, and EgPAT-T2 (Fig. 2). Some samples showed less than 100% cleavage efficiency, with three bands observed, including the uncut fragments. At the same time, the rest of the samples showed 100% cleavage efficiency with no uncut fragments.

**Fig. 3:**
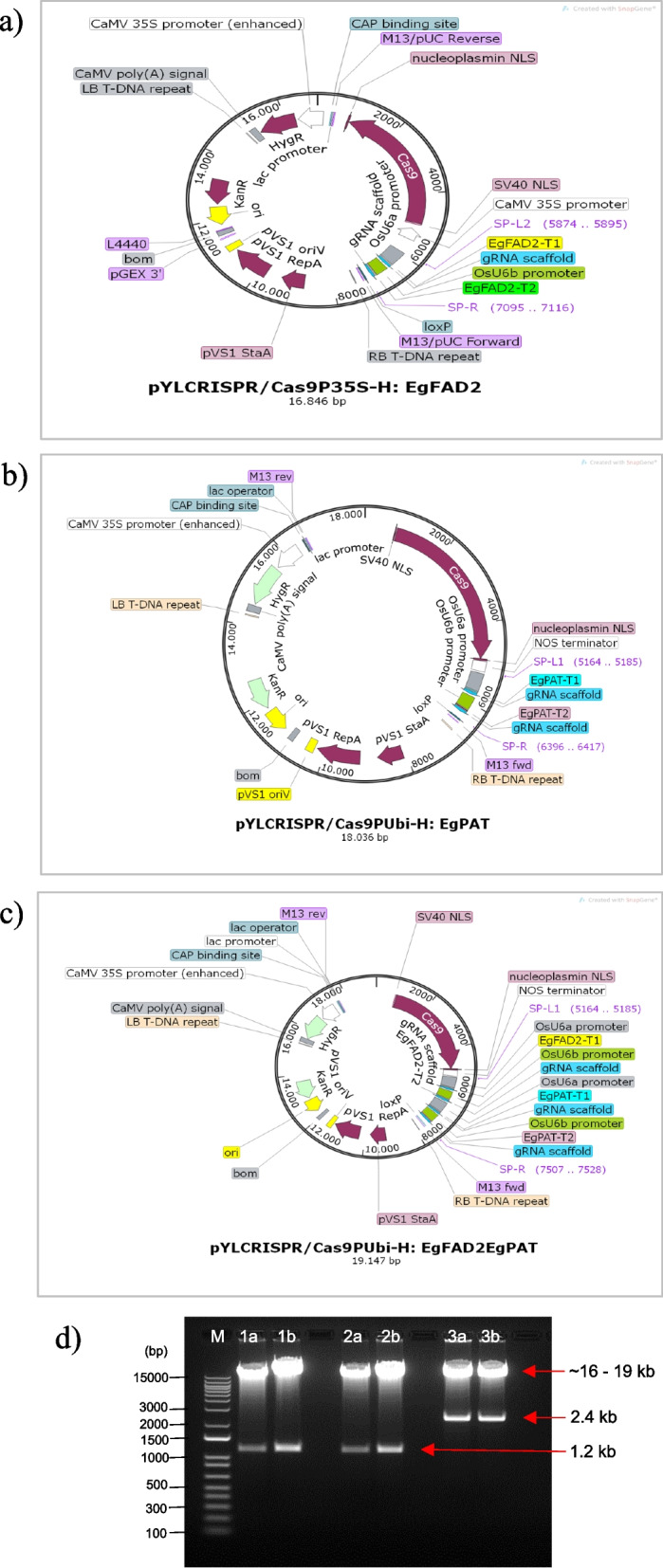
Schematic map for assembling two and four sgRNA expression cassettes into a pYLCRISPR/Cas9 vector (**a**) pYLCRISPR/Cas9P35S-H: EgFAD2 (16,846 bp), (**b**) pYLCRISPR/Cas9PUbi-H: EgPAT (18,036 bp), and (**c**) pYLCRISPR/Cas9PUbi-H: EgFAD2EgPAT (19,147 bp). T35S, cauliflower mosaic virus *35S* terminator; Hyg, hygromycin phosphotransferase gene; LB, left border of T-DNA; 2xP35S, the 35S promoter of the double cauliflower mosaic virus; Tnos, nopaline synthase gene terminator; P*Ubi*, maize ubiquitin Ubi1 promoter; Cas9, CRISPR-associated protein 9; T-right DNA’s border, abbreviated as RB. Rice (*Oryza sativa*) U6a promoter; Rice (*Oryza sativa*) U6b promoter; The GA-L and GA-R primers are used for Gibson assembly of sgRNA expression cassettes, while the flanking primers SP-L, SP-R, PB-L, and PB-R are employed to amplify the linked sgRNA expression cassettes. (**d**) *Asc*I digestion to ensure that the sgRNA expression cassettes are successfully inserted. Lanes 1a and 1b: pYLCRISPR/Cas9P35S-H: EgFAD2; Lanes 2a and 2b: pYLCRISPR/Cas9PUbi-H: EgPAT; Lanes 3a and 3b: pYLCRISPR/Cas9PUbi-H: EgFAD2EgPAT

A robust plant CRISPR/Cas9 vector system was used in this study. The vector system could efficiently assemble up to eight sgRNA expression cassettes into a single binary CRISPR/Cas9 vector [34]. This study assembled a maximum of four sgRNA expression cassettes into a linearised pYLCRISPR/Cas9 binary vector (Fig. 3). For assembling the sgRNA expression cassettes, two binary vectors, pYLCRISPR/Cas9P35S-H and pYLCRISPR/Cas9PUbi-H, each carrying a plant codon optimized *Cas9* gene (*Cas9*) under the control of the cauliflower mosaic virus 35S promoter (P*35S*) and the maize ubiquitin promoter (P*Ubi*), were used [34]. These vectors also contain hyg (-H) markers, which encode the *hygromycin phosphotransferase* gene. The sgRNAs were placed under the control of each *Oryza sativa* (*OsU6)* promoter in either the pYLsgRNA-OsU6a or pYLsgRNA-OsU6b expression vector [34]. The sgRNA expression cassettes, U6aT1FAD2 (629 bp), U6bT2FAD2 (564 bp), U6aT1PAT (629 bp), and U6bT2PAT (564 bp), were amplified by overlapping PCR and assembled into the binary vectors with the Gibson Assembly method (Gibson et al. 2009). Two vectors carrying two sgRNA expression cassettes, each targeting the *EgFAD2* gene (pLYCRISPRCas9P35S-H: EgFAD2) or the *EgPAT* gene (pLYCRISPRCas9PUbi-H: EgPAT), and one vector with the combination of all four sgRNA expression cassettes targeting both the *EgFAD2* and *EgPAT* genes, namely pLYCRISPRCas9PUbi-H: EgFAD2EgPAT, were successfully constructed (Fig. 3a, b, c). The three generated plasmids were confirmed with *Asc*I restriction endonuclease (Fig. 3d) and DNA sequencing (Fig. S2).

### CRISPR/Cas9-mediated genome editing of the EgFAD2 and EgPAT genes in transgenic oil palm

**Fig. 4:**
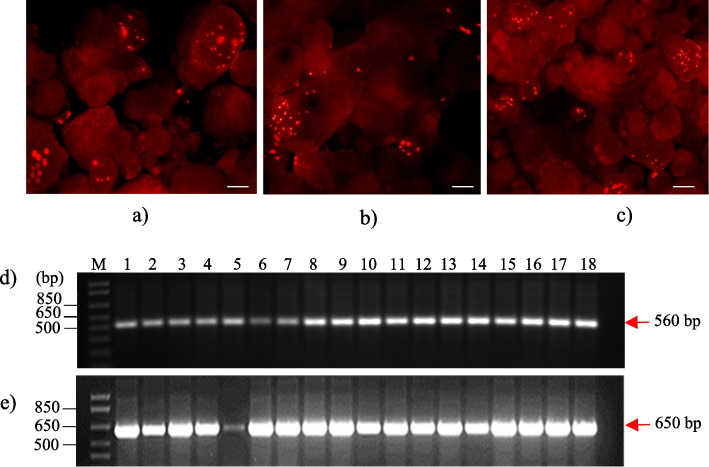
Molecular analysis of embryogenic calli in transgenic oil palm. Three days after co-bombardment of pAMdsRED with pLYCRISPRCas9P35S-H: EgFAD2 (**a**), pLYCRISPRCas9PUbi-H: EgPAT (**b**), and pLYCRISPRCas9PUbi-H: EgFAD2EgPAT (**c**), DsRED fluorescent spots on calli were observed. The scale bar is 200 µm. PCR analysis using cDNA (**d**) to detect the expression of *Cas9* with (**e**) Actin as an internal control to confirm the quality and integrity of the cDNAs. pLYCRISPRCas9P35S-H: EgFAD2, pLYCRISPRCas9PUbi-H: EgPAT, and pLYCRISPRCas9PUbi-H: EgFAD2EgPAT are found in lanes 1–6, 7–12, and 13–18. The results showed that *Cas9* gene expression was detected, as indicated by 560 bp PCR products. Lane M is a 1-kb plus DNA ladder (Invitrogen)

A proof-of-concept study utilizing oil palm protoplasts was conducted to test the multiplex CRISPR/Cas9 genome-editing system in oil palm. PEG-mediated transfection was used to deliver the constructed CRISPR/Cas9 vectors into oil palm protoplasts for transient expression. Meanwhile, biolistic and *Agrobacterium*-mediated methods were applied to deliver the vectors into oil palm embryogenic calli for stable transformations. RFP, a red fluorescent protein, was used as a visual marker to gauge the efficiency of biolistic-mediated transformation. Oil palm embryogenic calli were co-bombarded with pAMDsRED [35], a plasmid-carrying gene encoding *DsRED*. The red fluorescent signals from the expression of the *DsRED* gene were readily detected at 48 h after the transformation, which confirmed the successful delivery of CRISPR/Cas9 vectors into oil palm calli (Fig. 4a–c). Furthermore, the presence of *hyg* and *Cas9* genes was confirmed by PCR analysis (data not shown), and the expression of Cas9 was determined by RT-PCR analysis (Fig. 4d). This was performed to detect the expression of *Cas9* (Fig. 4d) in the transformed oil palm tissues. Meanwhile, PCR amplification of Actin (*EgAct* as an internal control) was also performed to check the quality and integrity of the cDNAs (Fig. 4e). The results showed that *Cas9* was expressed in most of the transformed oil palm tissues. However, the intensities of gel electrophoresis bands suggested different levels of Cas9 expression among different lines.

### Detection of mutations in transgenic oil palm samples by inference of CRISPR editing (ICE) analysis

**Fig. 5:**
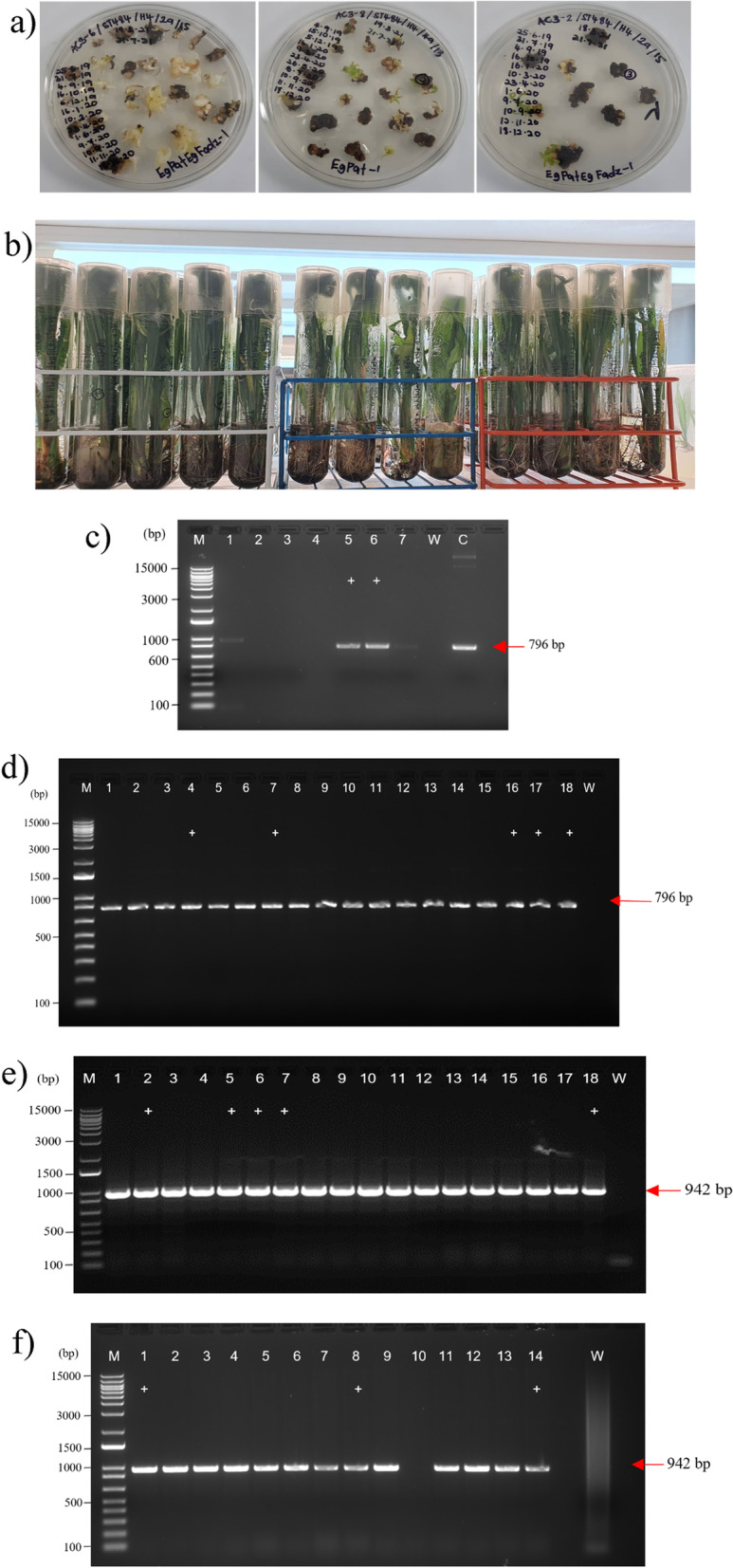
Confirmation of CRISPR/Cas9 transformation in oil palm. (**a**) Hygromycin selection of CRISPR/Cas9 transformed embryogenic calli and (**b**) plantlet regeneration. Gel electrophoresis of PCR amplicons using specific primers. (**c**) FAD2-R and FAD2-F primer sets were used to amplify PCR products from transfected protoplasts with pYLCRISPRCas9P35S-H: EgFAD2. Lanes 5 and 6 are positive samples; P5 and P6 are marked with a “ + .” (**d**) PCR products from embryogenic calli regenerated by *Agrobacterium*-mediated stable transformation with pYLCRISPR/Cas9PUbi-H: EgFAD2EgPAT using FAD2-F and FAD2-R primers. Positive samples of AC4 are found in lanes 4, 7, 16, 17, and 18. Positive samples of AC7, AC16, AC17, and AC18 are marked with a “ + .” (**e**) PCR products for embryogenic calli derived from *Agrobacterium*-mediated transformation with the pYLCRISPR/Cas9PUbi-H: EgFAD2EgPAT using EgPAT primer pairs. Lanes 2, 5, 6, 7, and 18 are positive samples of AC2, AC5, AC6, AC7, and AC18, respectively; and (**f**) PCR products for the regenerated embryogenic calli bombarded with the pYLCRISPRCas9PUbi-H: EgPAT plasmid using the EgPAT-F and EgPAT-R primers; lanes 1, 8, and 14 are positive samples of BC1, BC8, and BC14

For the detection of CRISPR/Cas9-induced mutations in transformed oil palm cells, 18 pools of protoplast samples transfected by PEG-mediated (P), 54 samples of embryogenic calli derived from *Agrobacterium*-mediated transformation (AC), and 144 samples derived from hygromycin-resistant embryogenic calli (BC) were randomly selected for analysis (Fig. 5a). Genomic DNA was extracted and subjected to PCR amplification. PCR analysis of pooled protoplast samples (Fig. 5c) and embryogenic calli regenerated by *Agrobacterium*-mediated stable transformation samples (Fig. 5d) revealed that a 796-bp fragment corresponding to the target region of the *EgFAD2* gene were successfully amplified, while a 942-bp fragment of the *EgPAT* gene were amplified from genomic DNA samples extracted from embryogenic calli derived from *Agrobacterium*-mediated transformation (Fig. 5e) and biolistic transformation (Fig. 5f). Targeted PCR amplicons from wild-type and putatively transgenic oil palm tissues were directly sequenced and analyzed by ICE (Inference of CRISPR Edits), consisting of seven and eight PCR positives for *EgFAD2* and *EgPAT* genes, respectively, that yielded a single band using primer pairs targeting *EgFAD2* and *EgPAT* genes (Lanes marked with a “ + ” in Fig. 5c–f). The ICE tool was developed by Synthego and uses Sanger sequencing data resulting from NHEJ of CRISPR-edited samples. The sequencing data file and the sgRNA, single or multiguide up to three sgRNAs per gene, were uploaded to the ICE software and were further analyzed to calculate the indel percentage (ICE score), knockout (KO) score, and *r*^2^ regression, showing the degree of alignment between the treated and control populations.

**Fig. 6:**
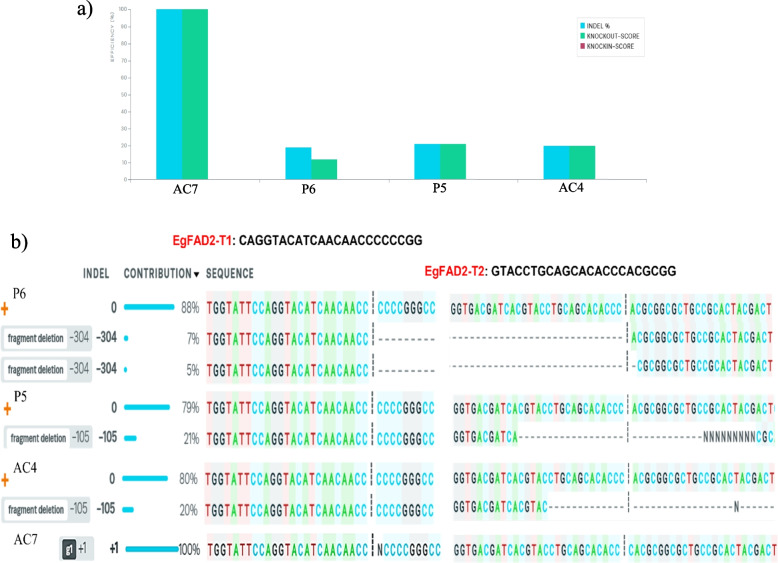
Genome editing in oil palm uses the inference of CRISPR Edits (ICE) to estimate the editing efficiency. The ICE indel profiles using dual EgFAD2 sgRNAs are represented graphically for two samples of edited protoplast cells transfected with pYLCRISPR/Cas9PUbi-H: EgFAD2 and two samples regenerated from *Agrobacterium-*mediated transformation with pYLCRISPR/Cas9PUbi-H: EgFAD2EgPAT. (**a**) The summary results for four samples that were edited by two sgRNAs, including indels and the percent of sequences that are putative knockouts (KO score). (**b**) The contributions tab shows indels and sequence lists from the dual-sgRNA-edited samples. The black vertical dashed lines represent the cleavage site matched with the site of the most upstream cut for the multiplex sample. The sequence plot represents mutations with fragment deletion and insertion, respectively. The contributions tab shows the inferred sequences and their relative proportions in an edited population, with the “ + ” symbol representing a wild type of sample

Mutations were detected at each target site in genomic samples derived from all vectors. For the EgFAD2 sgRNA target sites, four samples, AC7, P6, P5, and AC4, that consisted of mutations at both targeted sites, EgFAD2-T1 sgRNA and EgFAD2-T2 sgRNA, with indel percentages of 100%, 19%, 21%, and 20%, respectively (Fig. 6a). The sequence plot represents fragment deletion of EgFAD2-T1 sgRNA and EgFAD2-T2 sgRNA for P5 and P6 samples in oil palm edited protoplast cells transfected with pYLCRISPR/Cas9PUbi-H: EgFAD2, AC4 sample regenerated *Agrobacterium-*mediated explant transformed with pYLCRISPR/Cas9PUbi-H: EgFAD2EgPAT, and mutation with insertion of 1 bp for the AC7 sample (Fig. 6b). As shown in Fig. 6b, fragment deletions observed ranged from 105 to 304 bp. The deletions were initiated at the fourth nucleotide before the PAM site of EgFAD2-T1 sgRNA and three nucleotides before the PAM site of EgFAD2-T2 sgRNA. One of the edited samples from *Agrobacterium*-mediated transformed embryogenic calli (AC7) was found to be mono-allelic homozygous with an indel percentage of 100%. The other three samples were found heterozygous for mono-allelic, bi-allelic, and multi-allelic mutations (Table 1).

**Table 1:** Indel percentage, knockout scores, and mutation type in the transformed oil palm tissues predicted by ICE analysis

Target gene	SgRNA	Constructs	Sample	Indel (%)	Model fit (*R*^2^)	Knockout score	Mutant allele size (bp)	Mutation type
*EgFAD2*	EgFAD2-T1 EgFAD2-T2	pYLCRISPR/Cas9P35S-H: EgFAD2	P5	21	0.99	21	-105	Mono-allelic
		pYLCRISPR/Cas9P35S-H: EgFAD2	P6	19	0.94	12	-304, -304	Bi-allelic
		pYLCRISPR/Cas9PUbi-H: EgFAD2EgPAT	AC4	20	1	20	-105	Mono-allelic
		pYLCRISPR/Cas9PUbi-H: EgFAD2EgPAT	AC7	100	0.84	100	+ 1	Mono-allelic
	EgFAD2-T2	pYLCRISPR/Cas9PUbi-H: EgFAD2EgPAT	AC16	81	0.81	80	-20, -28, -23, -14, -12	Multi-allelic
		pYLCRISPR/Cas9PUbi-H: EgFAD2EgPAT	AC17	81	0.81	80	-20, -28, -23, -14, -12	Multi-allelic
		pYLCRISPR/Cas9PUbi-H: EgFAD2EgPAT	AC18	2	0.96	1	+ 6, -21	Bi-allelic
*EgPAT*	EgPAT-T1	pYLCRISPR/Cas9PUbi-H: EgPAT	BC1	3	0.94	3	-5, -10	Bi-allelic
		pYLCRISPR/Cas9PUbi-H: EgPAT	BC8	3	0.98	3	+ 4, -11, -10	Multi-allelic
		pYLCRISPR/Cas9PUbi-H: EgPAT	BC14	11	0.79	11	-1, + 13, -16	Multi-allelic
	EgPAT-T2	pYLCRISPR/Cas9PUbi-H: EgFAD2EgPAT	AC2	3	0.88	3	+ 2, -2	Bi-allelic
		pYLCRISPR/Cas9PUbi-H: EgFAD2EgPAT	AC5	1	0.93	1	-10	Mono-allelic
		pYLCRISPR/Cas9PUbi-H: EgFAD2EgPAT	AC6	2	0.91	2	+ 2	Mono-allelic
		pYLCRISPR/Cas9PUbi-H: EgFAD2EgPAT	AC7	12	0.75	6	+ 18, + 2, -38, -21, -13	Multi-allelic
		pYLCRISPR/Cas9PUbi-H: EgFAD2EgPAT	AC18	1	0.93	1	-10	Mono-allelic

The samples were labeled with “P” for protoplasts-, “AC” for *Agrobacterium*-, and “BC” for biolistic-mediated transformations

**Fig. 7:**
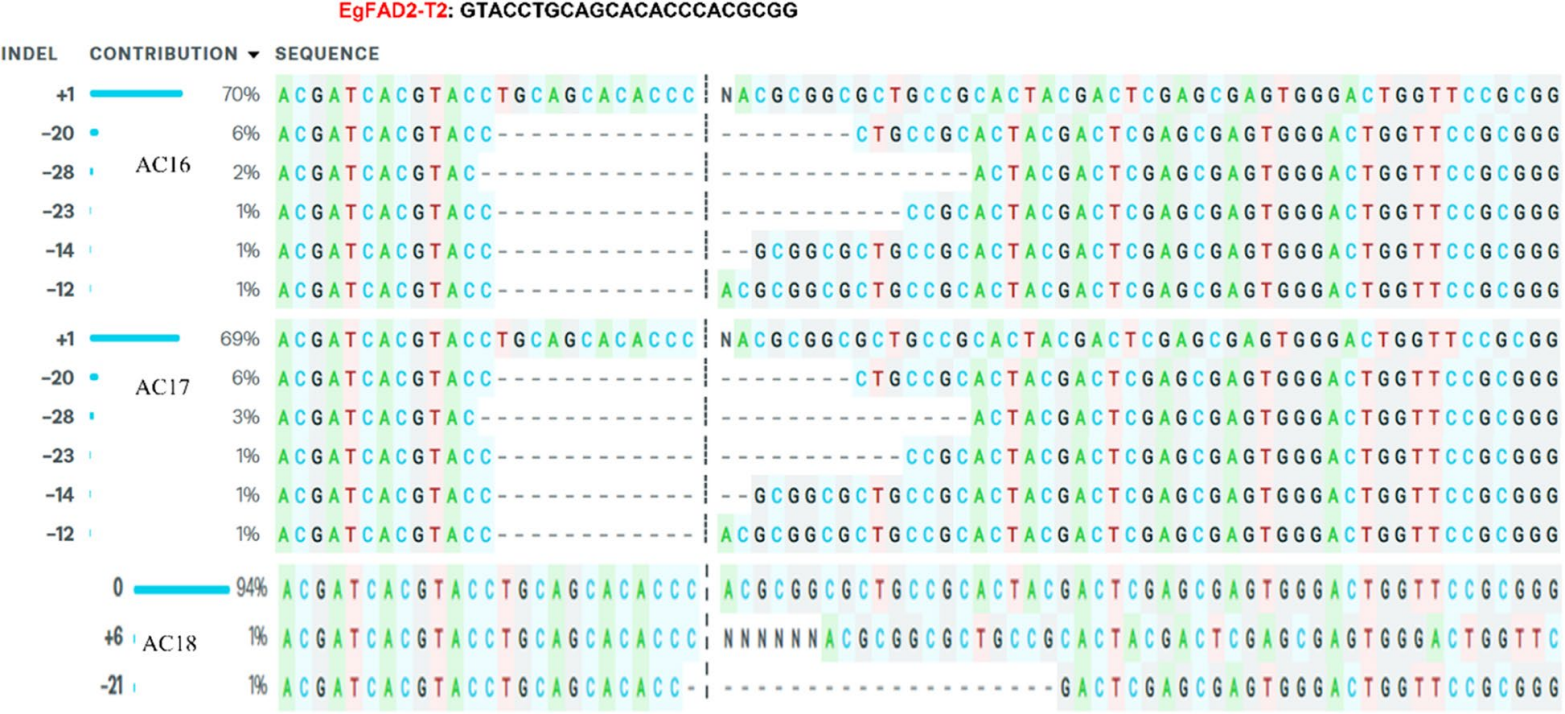
INDEL contribution results for edited samples determine the type of mutation (indel) and editing efficiency (contribution) with EgFAD2 sgRNA regenerated by *Agrobacterium*-mediated stable transformation with pYLCRISPR/Cas9PUbi-H: EgFAD2EgPAT. AC16, AC17, and AC18 samples shows indels in EgFAD2-T2 sgRNA. The sequence plot represents the ICE analysis. The black vertical dotted lines represent the cut sites, and “ + ” on the far left side denotes the control sample

However, we also identified mutagenesis at individual sgRNA target sites at EgFAD2-T2 sgRNA of samples regenerated by *Agrobacterium*-mediated stable transformation with pYLCRISPR/Cas9PUbi-H: EgFAD2EgPAT, with an editing efficiency of 81% observed in AC16 and AC17 samples (Fig. 7; Table 1). Both had multiple types of indels with five deletion events (− 20, − 28, − 23, − 14, and − 12 nt), while in AC18, knockout scores were 2% and + 6, − 21 indels (Fig. 7). The *r*^2^ regression results showed that EgFAD2 sgRNAs had *r*^2^ scores of 0.81 to 1, suggesting that 81 to 100% of the data variability was captured by ICE analysis. The KO scores ranged from 12 to 100, representing the proportion of cells with either a frameshift or indels that could contribute to a functional knockout of the target gene (Table 1).

**Fig. 8:**
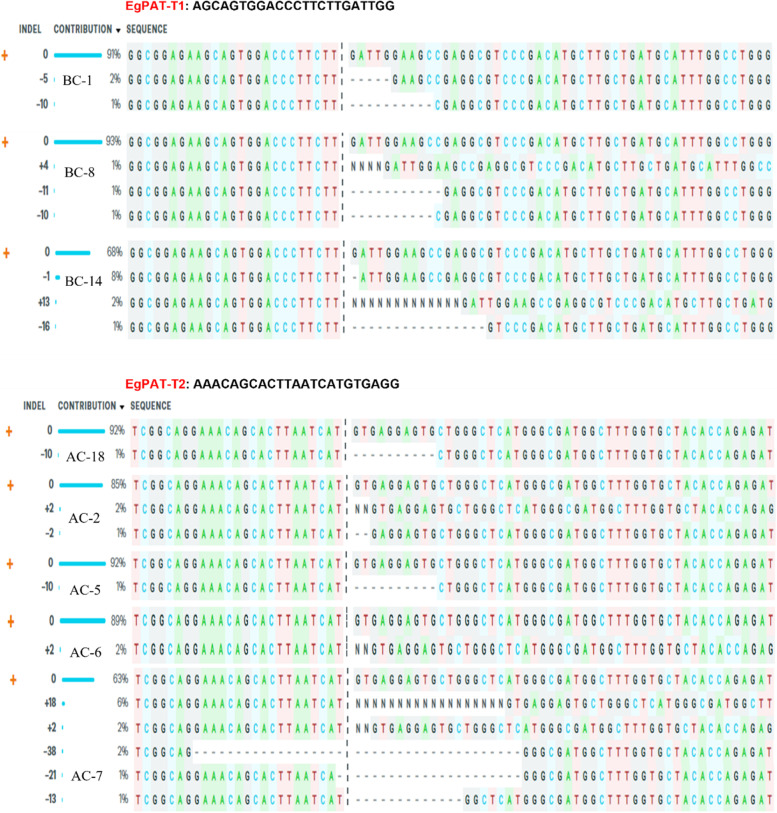
ICE results for amplified BC samples regenerated by biolistic transformation and AC samples regenerated by *Agrobacterium*-mediated transformation with pYLCRISPRCas9PUbi-H: EgPAT. The sequence plot represents the ICE analysis for EgPAT-T1 (BC-1, BC-8, and BC-14) and EgPAT-T2 (AC-18, AC-2, AC-5, AC-6, and AC-7) sgRNAs edited from oil palm embryogenic calli. The black vertical dotted lines represent the cut sites, and “ + ” on the far-left side denotes the control sample

Mutations were also detected at the EgPAT sgRNA target sites. However, indels were observed at individual targeted sites only. Three samples of bombarded calli (BC1, BC8, and BC14) showed mutations at EgPAT-T1 sgRNA with editing efficiencies of 3% in BC1 and BC8 samples and 11% in BC14 samples with three distinct editing patterns (− 1, + 13, and − 16 nt) (Fig. 8; Table 1). The BC1 sample showed − 10 and − 5 nt deletion, and the BC8 sample has indels with + 4 nt insertion and − 10 nt deletion. Five edited samples (AC2, AC5, AC6, AC7, and AC18) derived from *Agrobacterium*-mediated transformation were identified at the target 2 site, EgPAT-T2 sgRNA (Fig. 8). ICE analysis showed indel percentages of 1 to 10% and achieved *r*^2^ scores of 0.75 to 0.98, with knockout scores ranging from 1 to 11. The two targeted sites contained deletions ranging from 1 to 38 nt and insertions of 2 to 18 nt at the predicted DSB site (Table 1). The Sanger traces for the altered EgFAD2 and EgPAT target sites, and wild-type samples are displayed on the ICE distributions and traces tab. The size of the insertion or deletion and the percentage of sequences that contain it are displayed on the graph (Figs. S3, S4 and S5).

Interestingly, multiple gene modifications were detected in individual samples of oil palm calli derived from *Agrobacterium*-mediated transformation. Two samples, AC7 and AC18, showed mutations at both targeted genes, derived from a vector carrying four sgRNAs. In the AC7 sample, mutations were found at both sgRNA (EgFAD2-T1 and EgFAD2-T2) targeted sites and one site of EgPAT (T2). Mutations were only found in the AC18 sample at each targeted site in each targeted gene, EgFAD2-T2, and EgPAT-T2. This result shows that the multiplex CRISPR/Cas9 system can be exploited to precisely edit oil palm genomic sequences and create multisite knockout mutations.

This study demonstrated the mutation rates of four target sites for two genes in transformed oil palm tissues by three delivery systems: namely, biolistic, PEG, and *Agrobacterium*-mediated transformation (Table 2). The mutation rate of EgFAD2 sgRNAs in oil palm protoplasts transfected by PEG-mediated transformation was 27%, whereas it was 28% in oil palm embryogenic calli regenerated by *Agrobacterium*-mediated transformation. No mutation was found in bombarded oil palm embryogenic calli targeting EgFAD2 sites. On the other hand, the EgPAT sgRNA mutation rate of 28% and 6% was observed for embryogenic calli derived from *Agrobacterium* and biolistic-mediated transformation, respectively (Table 2). In this study, CRISPR/Cas9 delivery into oil palm seems more efficient via *Agrobacterium*-mediated transformation than via biolistic.

**Table 2:** The mutation rate of the four target sites in transformed oil palm tissues by biolistic, PEG, and *Agrobacterium*-mediated transformations analyzed using PCR and Sanger sequencing

Transformation method	Target gene	Constructs	No. of examined samples	No. of mutated samples	Mutation rate (%)	Mutants
*Agrobacterium*	*EgFAD2*	pYLCRISPR/Cas9PUbi-H: EgFAD2	18	5	28	2 (Double sgRNAs EgFAD2_T1T2) 3 (Single sgRNA_EgFAD2_T2)
	*EgPAT*	pYLCRISPR/Cas9PUbi-H: EgPAT	18	5	28	5 (Single sgRNA_EgPAT_T2)
	*EgFAD2EgPAT*	pYLCRISPR/Cas9PUbi-H: EgFAD2EgPAT	18	2	11	2 (Multigene: EgFAD2_T2, EgPAT_T2)
	Total		54			
PEG	*EgFAD2*	pYLCRISPR/Cas9P35S-H: EgFAD2	6	2	33	2 (Double sgRNAs_EgFAD2_T1T2)
	*EgPAT*	pYLCRISPR/Cas9PUbi-H: EgPAT	7	0	0	-
	*EgFAD2EgPAT*	pYLCRISPR/Cas9PUbi-H: EgFAD2EgPAT	5	1	20	1 (Single EgFAD2_T1)
	Total		18			
Biolistic	*EgFAD2*	pYLCRISPR/Cas9P35S-H: EgFAD2	48	0	0	-
	*EgPAT*	pYLCRISPR/Cas9PUbi-H: EgPAT	48	3	6	3 (Single sgRNA_EgPAT_T1)
	*EgFAD2EgPAT*	pYLCRISPR/Cas9PUbi-H: EgFAD2EgPAT	48	0	0	-
	Total		144			

Mutation rate (%) = number of samples with mutations/number of total transgenic samples. Mutations were analysed by ICE

## Discussion

Targeting the *EgFAD2* and *EgPAT* genes in oil palm using the CRISPR/Cas9 system is expected to generate knockout mutant samples with a higher oleic acid content than the wild type. Furthermore, the ability of the multiplex CRISPR/Cas9 system to target multiple genomic sites using a single vector was evaluated in this study. Two sgRNAs for each target gene were designed to improve the probability of fragment deletions and increase the mutation rate of target genes. The genomic DNA obtained from transformed samples was Sanger sequenced, and the editing efficiency was analyzed. Despite low transformation efficiency rates of 0.7% for *Agrobacterium*-mediated transformation [29], 1–1.5% for biolistic methods [6], and 4.76% for protoplast PEG-mediated transfection [28], the efficiency of multiplex CRISPR/Cas9 with designed sgRNAs successfully introduced mutations into the oil palm.

We observed three types of modifications: mono-allelic, bi-allelic, and multi-allelic editing-based mutations between the two target genes. The PAM, which is usually the NGG sequence next to sgRNA in target DNA, is recognized by the Cas9 nuclease, which causes DSBs between the PAM’s third and fourth nucleotides. Among the four sgRNAs designed, EgFAD2-T1 and EgFAD2-T2 targeting the *EgFAD2* gene showed a higher editing efficiency than both EgPAT-T1 and EgPAT-T2 targeting the *EgPAT* gene. Our findings suggest that sgRNA with a high GC content has a higher mutation rate. EgFAD2 sgRNAs with 60% and 65% yielded indel rates ranging from 19 to 100%, whereas sgRNAs with 50% and 35% yielded indel rates ranging from 1 to 12% (Table S1). This indicates that sgRNAs with high GC content improve editing efficiency in oil palm. It was previously reported that GC content might influence the sgRNA’s binding capacity to its target and affect the activity of sgRNAs, resulting in increased or reduced editing efficiency [36, 37]. Early studies reported that the secondary structures of sgRNAs are important for Cas9/sgRNA effectiveness [38–40]. Based on their secondary structure, the efficacy of each gRNA, accessibility at a specific position for Cas9 binding, and the presence of stem loops were all predicted. The transfected protoplasts by PEG-mediated transformation and the calli infected by *Agrobacterium*-mediated transformation were able to generate a large deletion fragment of 304 nt and 105 nt, respectively, between two sites of EgFAD2-T1 sgRNA and EgFAD2-T2 sgRNA. This showed that this multiplex genome-editing platform could create large fragment deletions in oil palm. However, low editing efficiency through biolistic transformation was obtained. Therefore, it is necessary to improve delivery efficiency and establish a method for screening and improving genome-editing efficiency. Additionally, we also found that editing efficiency depends on the appropriate starting material and chosen delivery methods. Other than that, improving the oil palm transformation and regeneration procedure is critical for achieving a higher mutation ratio.

Previous studies have revealed that the oleic acid content was increased in CRISPR/Cas9-edited soybeans from 17.10 to 73.50% with a concomitant decrease in linoleic acid of 12.23% [20]. In tobacco, the content of oleic acid increased from 11 to 79%, while linoleic acid decreased to 7% from 72% [18]. In pennycress, the content of oleic acid increased to 91% compared to 12% in the wild type, while linoleic acid and linolenic acid decreased to less than 1% from 18% and less than 3% from 12%, respectively [16]. Therefore, it would be valuable to investigate whether targeted editing of these two *EgFAD2* and *EgPAT* genes could lead to significant oil content and fatty acid composition changes, mainly an increase in the oleic acid content in transgenic mutant oil palm samples. However, no fatty acid analysis could be performed now, and further analysis of the fatty acid content will be investigated during the oil palm fruit development stage. This is expected to have greater transcript levels of fatty acid biosynthesis enzymes. Nevertheless, the ICE from Sanger trace data could be used to overcome the difficulty of oil palm mutant screening, which is time-consuming, high-cost, and laborious. This is a promising tool heavily associated with next-generation sequencing [32].

Recently, Yeap et al. [13] and Jamaludin et al. [41] reported the CRISPR/Cas9 genome-editing system in oil palm, targeting the *EgPDS* gene that is involved in the carotenoid biosynthesis pathway. The *PDS* gene is a popular marker that has been extensively used in a new plant system as a proof of concept to quickly demonstrate the feasibility of CRISPR/Cas9 in oil palm. Yeap et al. [13] demonstrated that disruption of the *phytoene desaturase* (*PDS*) gene leads to photobleaching, the albino phenotype, and dwarfism, which would allow easier screening of mutated lines. They used oil palm immature embryos as starting material and propagated them through direct embryogenesis. However, the chimeric phenotype was observed in the transgenic, edited palms. Therefore, oil palm embryogenic calli were used in this study as the target tissue to knock out the *EgFAD2* and *EgPAT* genes. It has been reported that using embryogenic calli as starting material is more genetically stable and results in minor genetic variations in regenerated plants, which are more acceptable for commercial purposes [42–44].

Although with a low efficiency of up to 33% across the 6 transfected protoplasts and an average-editing efficiency of 18% across the 198 transformed embryogenic calli, our findings suggest that the CRISPR/Cas9 system was successfully used to target the *EgFAD2* and *EgPAT* genes in oil palm. The low-editing efficiency could be because the oil palm genome contains multiple copies of the endogenous *EgFAD2* and *EgPAT* genes, which resulted in low expression of the target gene. As an alternative in the future, modifications in specific *cis*-regulatory elements and promoter region removal are expected to increase the CRISPR/Cas9 genome-editing specificity and efficiency in oil palm. The next possible CRISPR/Cas9 system using the Cas9/sgRNA complex or ribonucleoprotein (RNP) will also be investigated. RNPs will reduce the possibility of off-target modifications and alleviate public concerns about genetically modified organisms, with the added benefit of generating transgene-free genome editing, which is expected to gain public acceptance.

## Conclusion

Demand for high levels of oleic acid is growing due to its versatility in industrial applications and for nutritional purposes. To meet rising demand, recent research has focused on the use of multiplex CRISPR/Cas9 genome-editing technology, which has the potential to produce higher oleic palm oil with lower saturated fatty acid content. We successfully generated knockout mutants with large and small deletions within the targeted regions by designing multiple sgRNAs targeting the *EgFAD2* and *EgPAT* genes. Two vectors carrying two sgRNA expression cassettes, each targeting the *EgFAD2* gene or the *EgPAT* gene, and one vector with the combination of all four sgRNA expression cassettes targeting both the *EgFAD2* and *EgPAT* genes, were successfully constructed. The presence of *hyg* and *Cas9* genes was confirmed by PCR analysis, and the expression of Cas9 was detected by RT-PCR analysis. CRISPR/Cas9 delivery into oil palm seems more efficient via *Agrobacterium*-mediated transformation than via biolistic. Due to oil palm being a perennial crop with a more than 25-year life cycle, the fatty acid content analysis, on the other hand, can only be done during the development stage of the oil palm fruit, the stage in which both genes are expected to be highly expressed. Nevertheless, these findings add to the preliminary but promising evidence that CRISPR/Cas9 genome editing can be used in oil palm.

## Supplementary Information

**Additional file 1: Fig. S1.** Secondary structures of all oil palm sgRNA transcripts of EgFAD2 and EgPAT target sgRNAs. **Fig. S2.** Sequencing results for plasmids a) pYLCRISPR/Cas9P35S-H: EgFAD2, and b) pYLCRISPR/Cas9PUbi-H: EgPAT containing two sgRNA expression cassettes for EgFAD2 and EgPAT target sites driven by Oryza sativa U6a promoter (T1) and U6b promoter (T2). **Fig. S3.** ICE distributions and traces tab for edited EgFAD2 target sites. (a) The ICE "Traces" shows percentage indel on the left histogram, and the discordance plot on the right displays the alignment between the edited sample in green and the wild type in orange. The EgFAD2 sgRNA1 and sgRNA2 regions in P5 cells show a knock-out (KO) score of 21, in P6 cells show a KO score of 19, in AC4 cells show a KO score of 20, and in AC7 cells show a KO score of 100. (b) Chromatograms of Cas9-edited genomic DNA containing dual sgRNA and a control trace. Cas9 target cut sites are represented by black vertical dashed lines; the guide sequence is underlined; and the sequence of the protospacer adjacent motif (PAM) is marked with a dotted red line. **Fig. S4.** Chromatograms of Cas9-edited genomic DNA containing EgFAD2-T2 sgRNA and a control trace for lines AC16, AC17 and AC18. Cas9 target cut sites are represented by black vertical dashed lines; the guide sequence is underlined; and the sequence of the protospacer adjacent motif (PAM) is marked with a dotted red line. **Fig. S5.** ICE traces tab of Cas9-edited genomic DNA containing EgPAT sgRNAs and a control trace for lines derived from bombarded (BC1, BC8 and BC14) and Agrobacterium mediated transformation (AC18, AC2, AC5, AC6 and AC7). Cas9 target cut sites are represented by black vertical dashed lines; the guide sequence is underlined; and the sequence of the protospacer adjacent motif (PAM) is marked with a dotted red line. **Table S1.** sgRNA selection of EgFAD2 and EgPAT genes. **Table S2.** Primers used in this study.

## Data Availability

The authors declare that all data generated or analyzed in this study are included in the article.

## Abbreviations

*Cas9*: CRISPR-associated protein 9
*CRISPR*: Clustered regularly interspaced short palindromic repeats
*FAD2*: Oleoyl-CoA desaturase
*PAT*: Palmitoyl-ACP-thioesterase
*Eg**FAD2*: *E. guineensis* FAD2
*EgPAT*: *E. guineensis* PAT
*PAM*: Protospacer adjacent motif
*PEG*: Polyethylene glycol
*sgRNA*: Single-guide RNA
*EgPDS*: *E.* *guineensis* *phytoene desaturase*
*EgBRI1*: *E. **guineensis* *brassinosteroid**-insensitive 1*
