# Supplementary material for: Multiplex CRISPR/Cas9 gene-editing platform in oil palm targeting mutations in EgFAD2 and EgPAT genes
Source: J Genet Eng Biotechnol. 2023 Jan 11;21:3. doi: 10.1186/s43141-022-00459-5 (PMC9834484; doi:10.1186/s43141-022-00459-5)
Supplement: Supplementary file 1 — Additional file 1: Fig. S1. Secondary structures of all oil palm sgRNA transcripts of EgFAD2 and EgPAT target sgRNAs. Fig. S2. Sequencing results for plasmids a) pYLCRISPR/Cas9P35S-H: EgFAD2, and b) pYLCRISPR/Cas9PUbi-H: EgPAT containing two sgRNA expression cassettes for EgFAD2 and EgPAT target sites driven by Oryza sativa U6a promoter (T1) and U6b promoter (T2). Fig. S3. ICE distributions and traces tab for edited EgFAD2 target sites. (a) The ICE "Traces" shows percentage indel on the left histogram, and the discordance plot on the right displays the alignment between the edited sample in green and the wild type in orange. The EgFAD2 sgRNA1 and sgRNA2 regions in P5 cells show a knock-out (KO) score of 21, in P6 cells show a KO score of 19, in AC4 cells show a KO score of 20, and in AC7 cells show a KO score of 100. (b) Chromatograms of Cas9-edited genomic DNA containing dual sgRNA and a control trace. Cas9 target cut sites are represented by black vertical dashed lines; the guide sequence is underlined; and the sequence of the protospacer adjacent motif (PAM) is marked with a dotted red line. Fig. S4. Chromatograms of Cas9-edited genomic DNA containing EgFAD2-T2 sgRNA and a control trace for lines AC16, AC17 and AC18. Cas9 target cut sites are represented by black vertical dashed lines; the guide sequence is underlined; and the sequence of the protospacer adjacent motif (PAM) is marked with a dotted red line. Fig. S5. ICE traces tab of Cas9-edited genomic DNA containing EgPAT sgRNAs and a control trace for lines derived from bombarded (BC1, BC8 and BC14) and Agrobacterium mediated transformation (AC18, AC2, AC5, AC6 and AC7). Cas9 target cut sites are represented by black vertical dashed lines; the guide sequence is underlined; and the sequence of the protospacer adjacent motif (PAM) is marked with a dotted red line. Table S1. sgRNA selection of EgFAD2 and EgPAT genes. Table S2. Primers used in this study. [file 43141_2022_459_MOESM1_ESM.docx]

**Supplementary Figures** **and Tables**


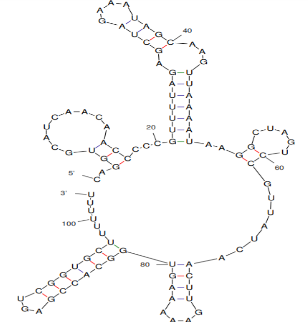

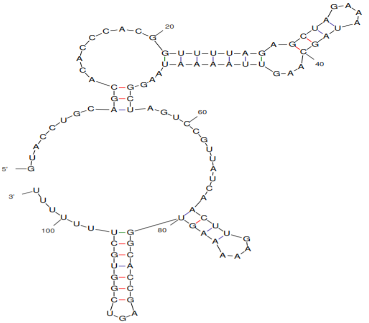

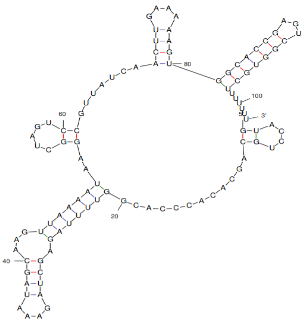

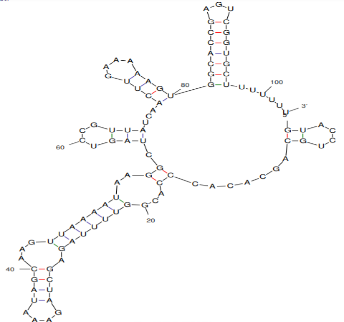


EgFAD2-T1 EgFAD2-T2a EgFAD2-T2b EgFAD2-T2c


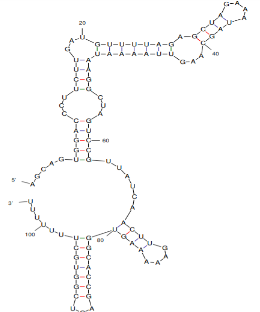

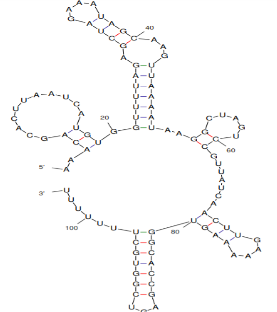

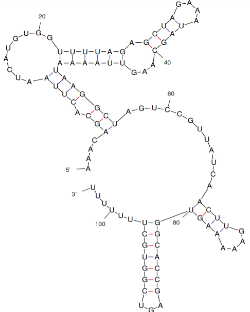

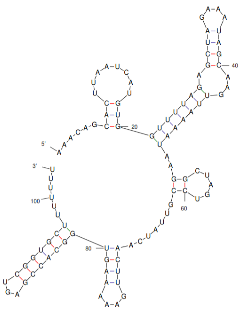

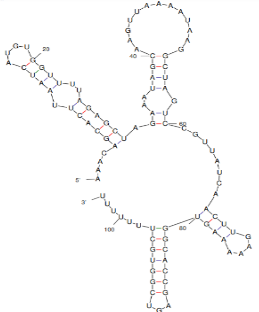


EgPAT-T1 EgPAT-T2a EgPAT-T2b EgPAT-T2c EgPAT-T2d


**Fig. S1.**  Secondary structures of all oil palm sgRNA transcripts of EgFAD2 and EgPAT target sgRNAs

a)

b)

BLUE: OsU6a Promoter PURPLE: EgFAD2-T1 sgRNA RED: EgFAD2-T2 sgRNA

GREY: OsU6b Promoter DARK PURPLE: EgPAT-T1 sgRNA YELLOW: EgPAT-T2 sgRNA

**Fig. S2.**  Sequencing results for plasmids a) pYLCRISPR/Cas9P35S-H: EgFAD2, and b) pYLCRISPR/Cas9PUbi-H: EgPAT containing two sgRNA expression cassettes for *EgFAD2* and *EgPAT* target sites driven by *Oryza sativa* U6a promoter (T1) and U6b promoter (T2).

P5

P6

AC4

AC7

a)


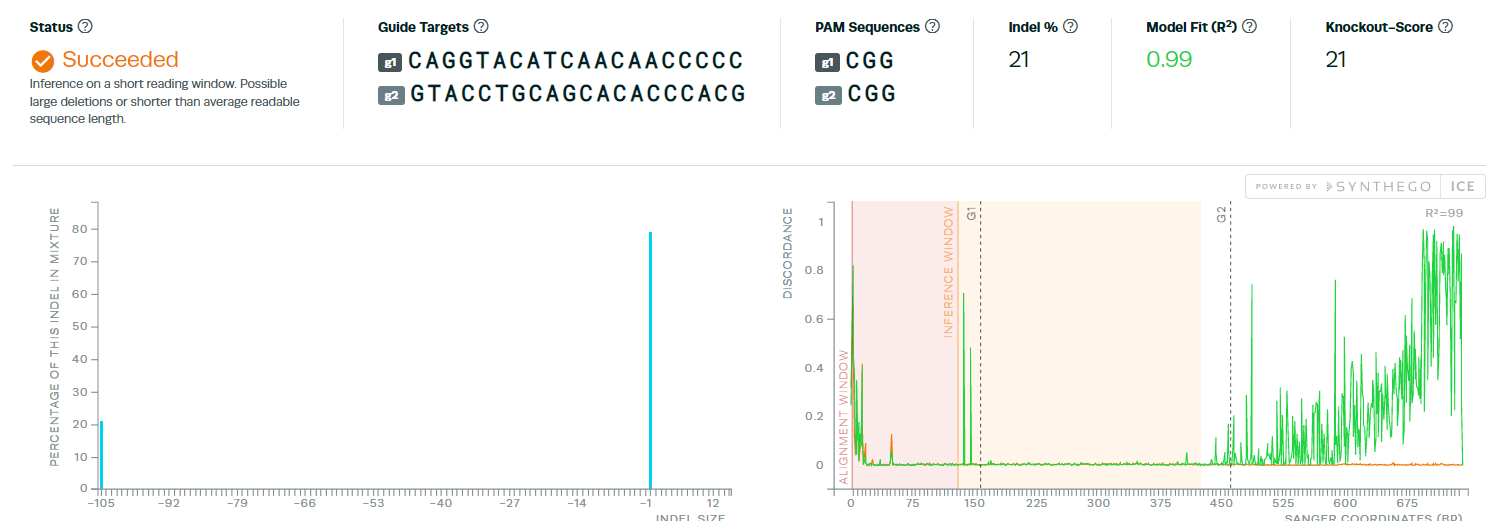

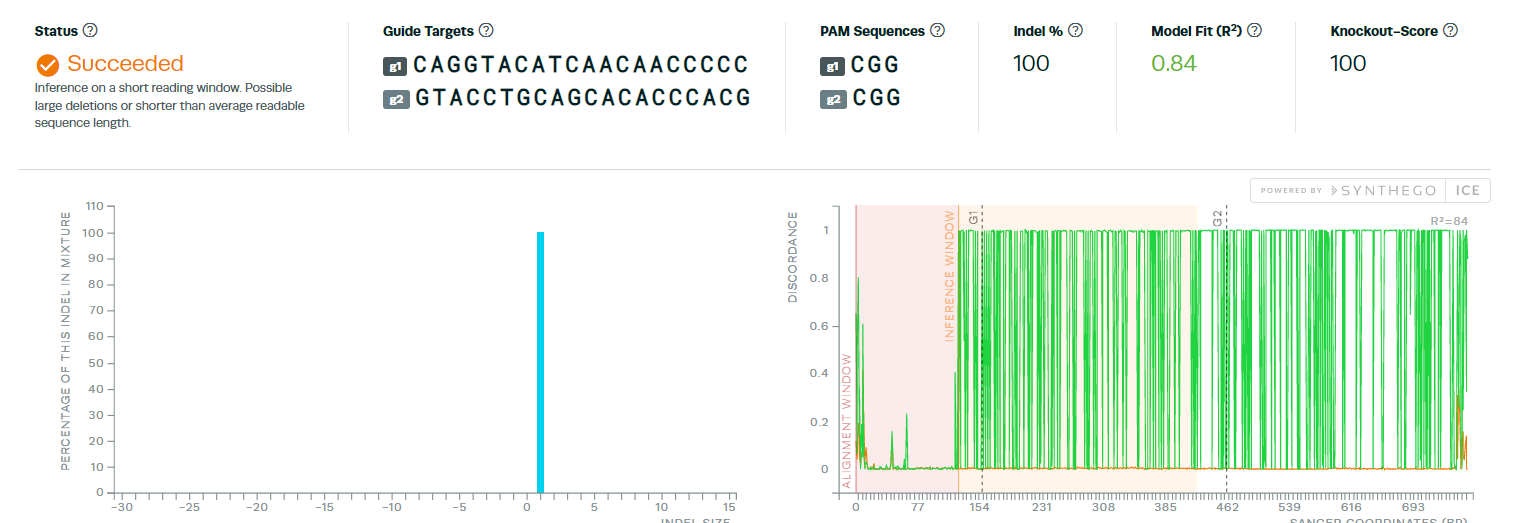

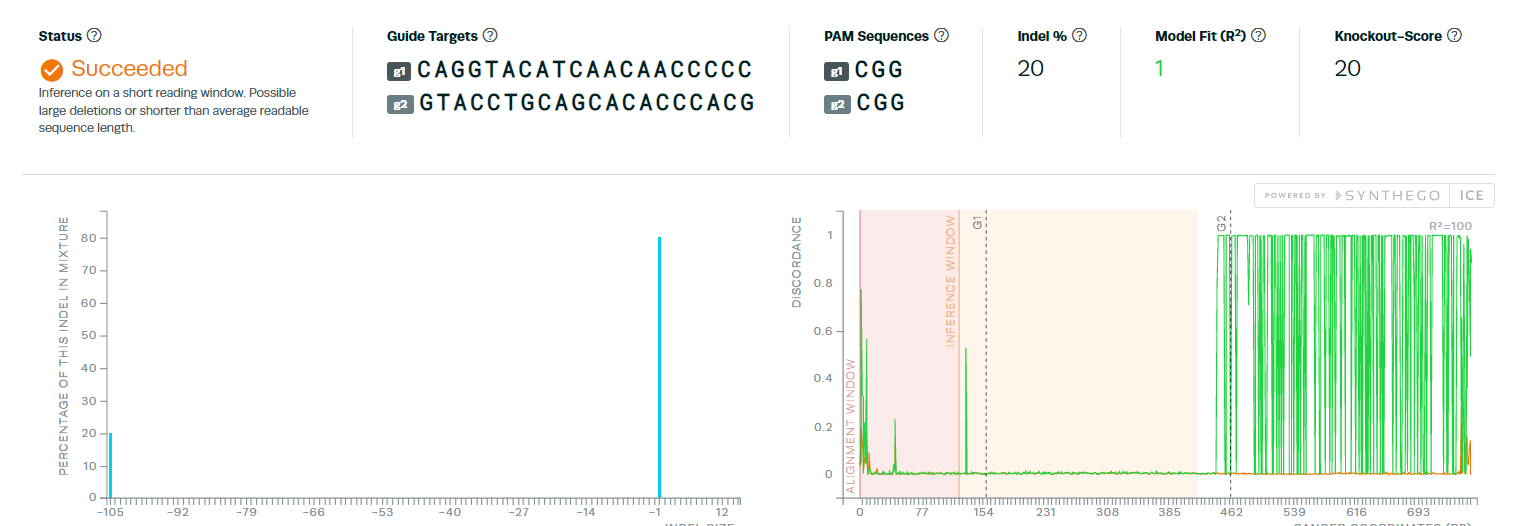

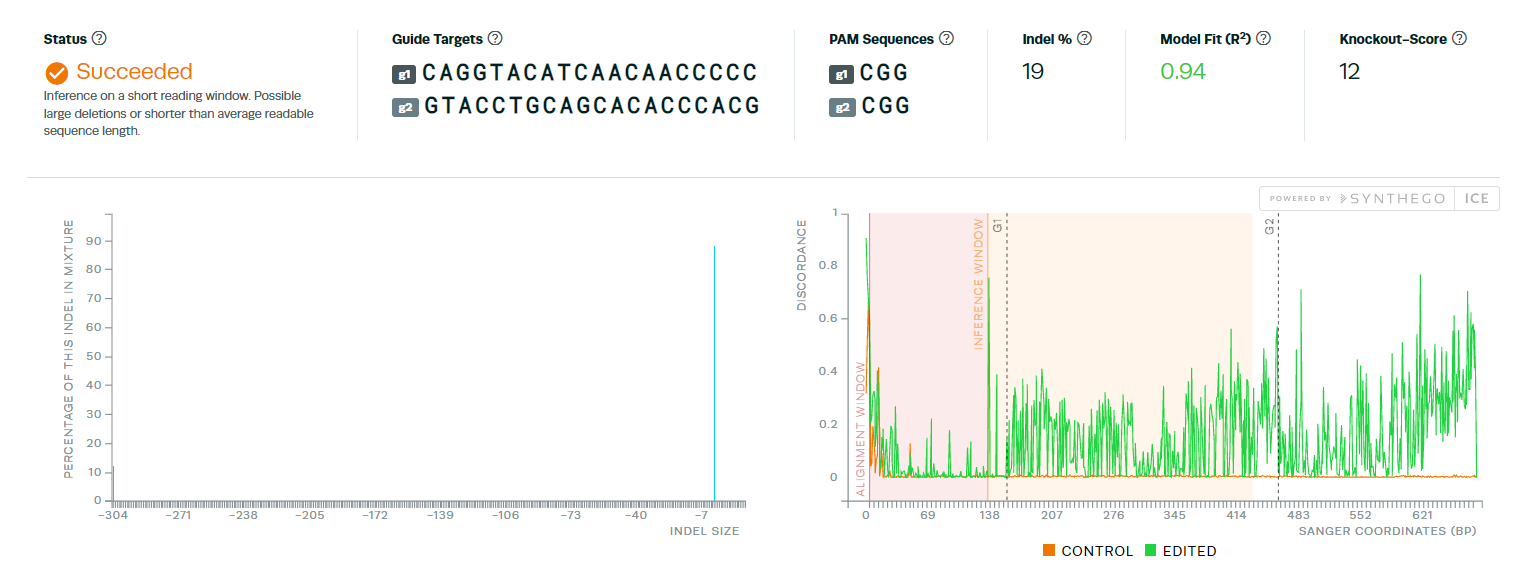


b)


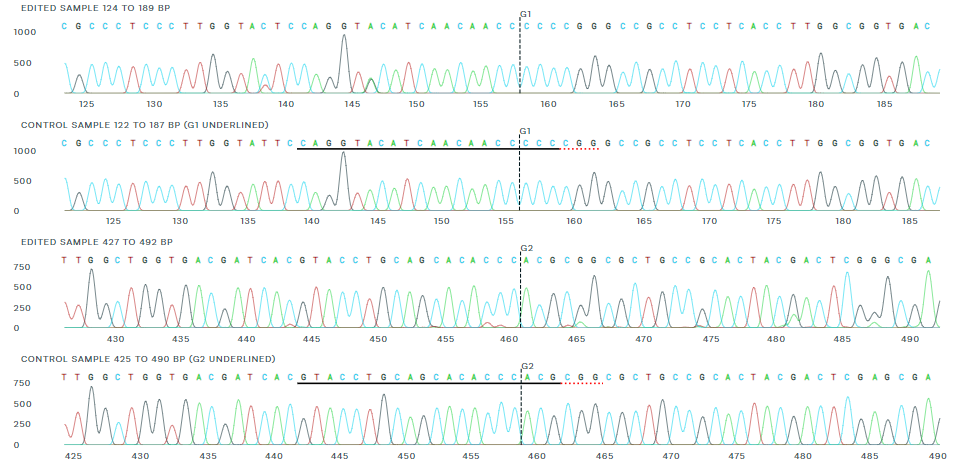

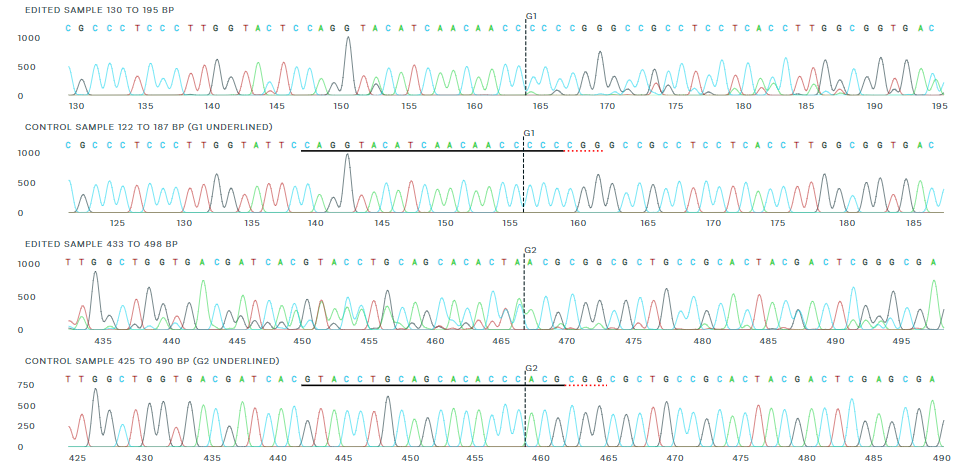

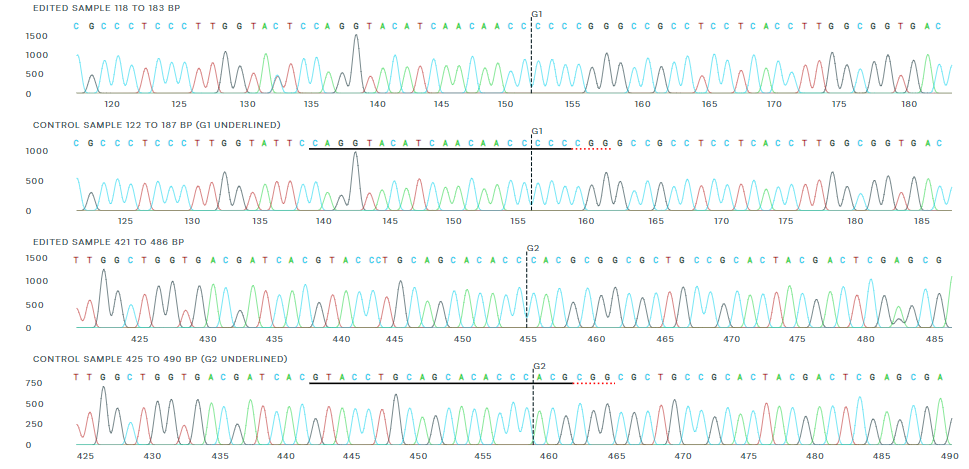

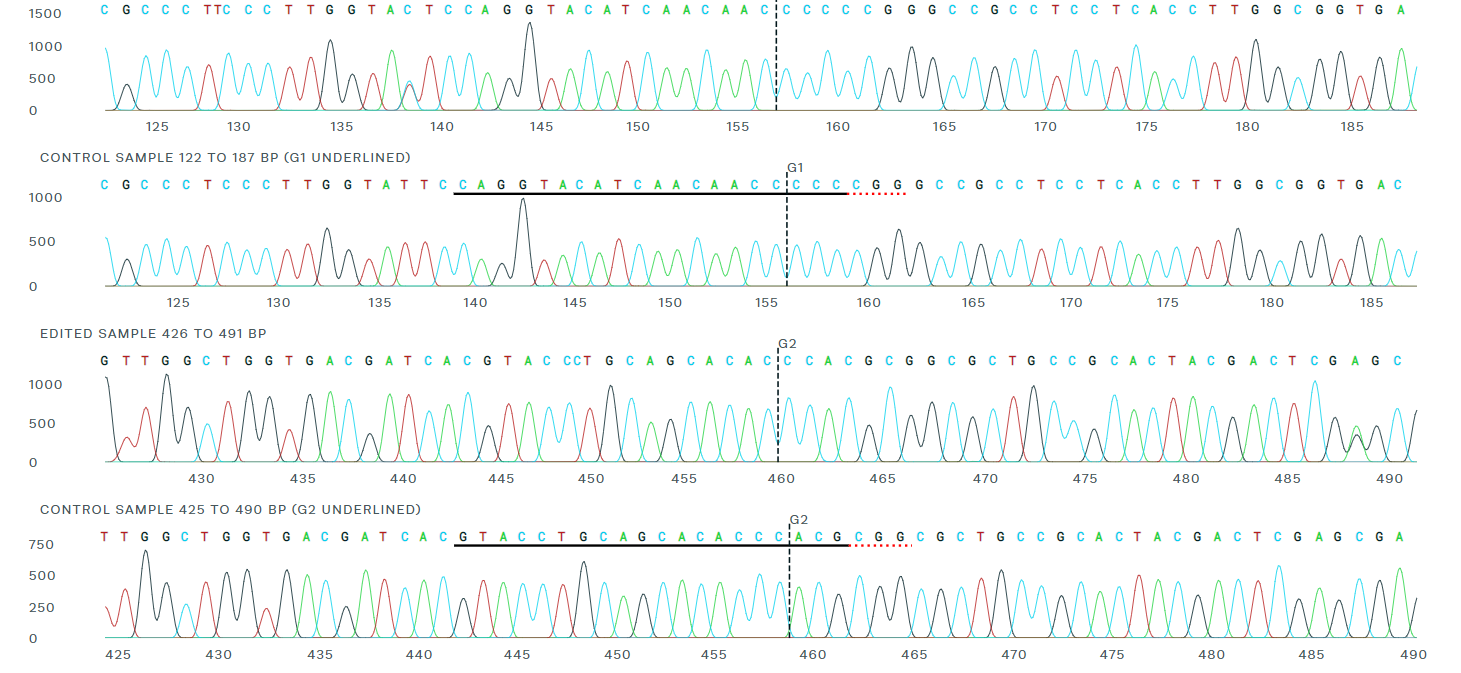


AC4

P5

P6

AC7

**Fig. S3.** ICE distributions and traces tab for edited EgFAD2 target sites. (a) The ICE "Traces" shows percentage indel on the left histogram, and the discordance plot on the right displays the alignment between the edited sample in green and the wild type in orange. The EgFAD2 sgRNA1 and sgRNA2 regions in P5 cells show a knock-out (KO) score of 21, in P6 cells show a KO score of 19, in AC4 cells show a KO score of 20, and in AC7 cells show a KO score of 100. (b) Chromatograms of Cas9-edited genomic DNA containing dual sgRNA and a control trace. Cas9 target cut sites are represented by black vertical dashed lines; the guide sequence is underlined; and the sequence of the protospacer adjacent motif (PAM) is marked with a dotted red line.


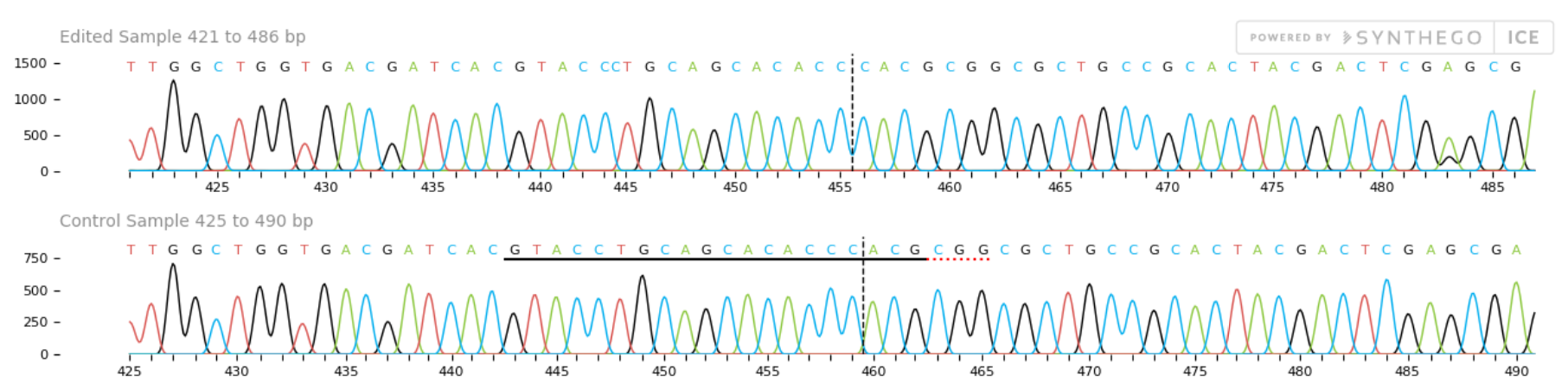


AC16


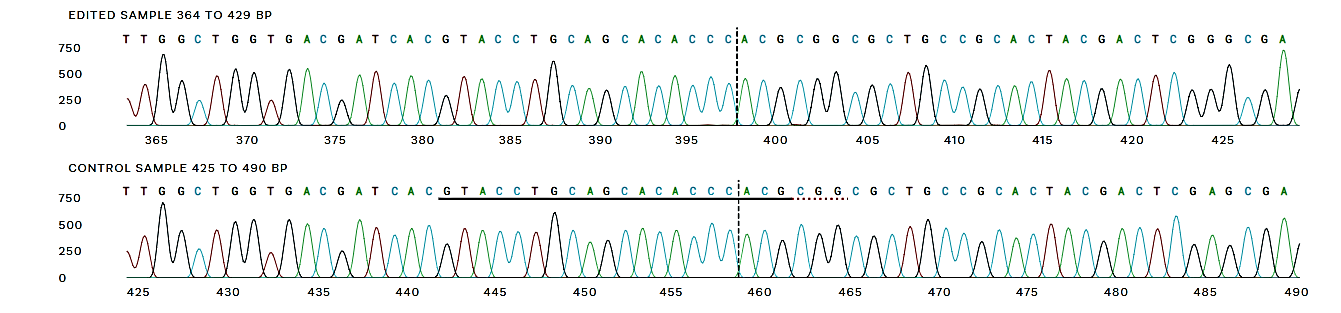

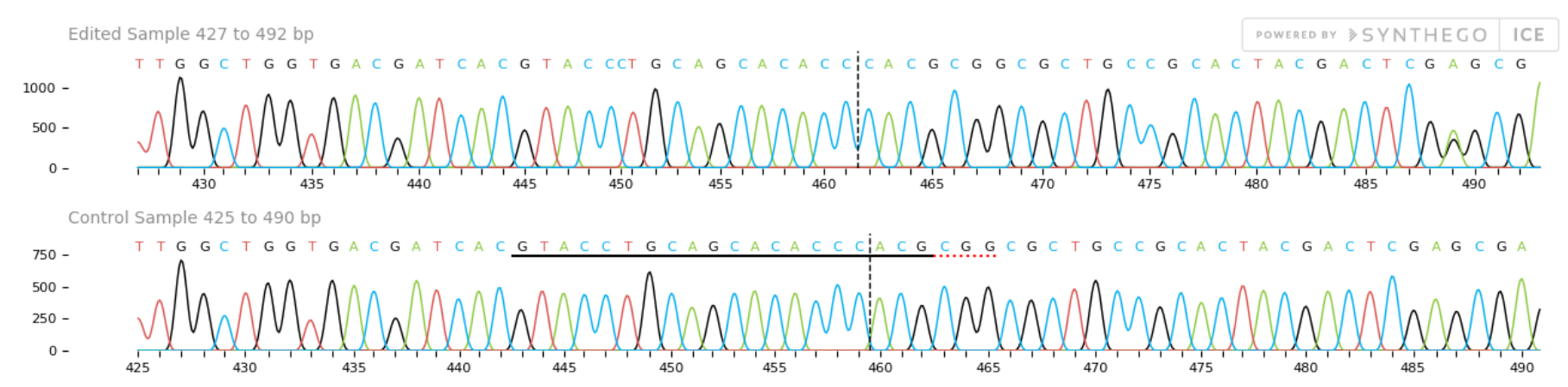


AC17

AC18

**Fig. S4.** Chromatograms of Cas9-edited genomic DNA containing EgFAD2-T2 sgRNA and a control trace for lines AC16, AC17 and AC18. Cas9 target cut sites are represented by black vertical dashed lines; the guide sequence is underlined; and the sequence of the protospacer adjacent motif (PAM) is marked with a dotted red line.

**
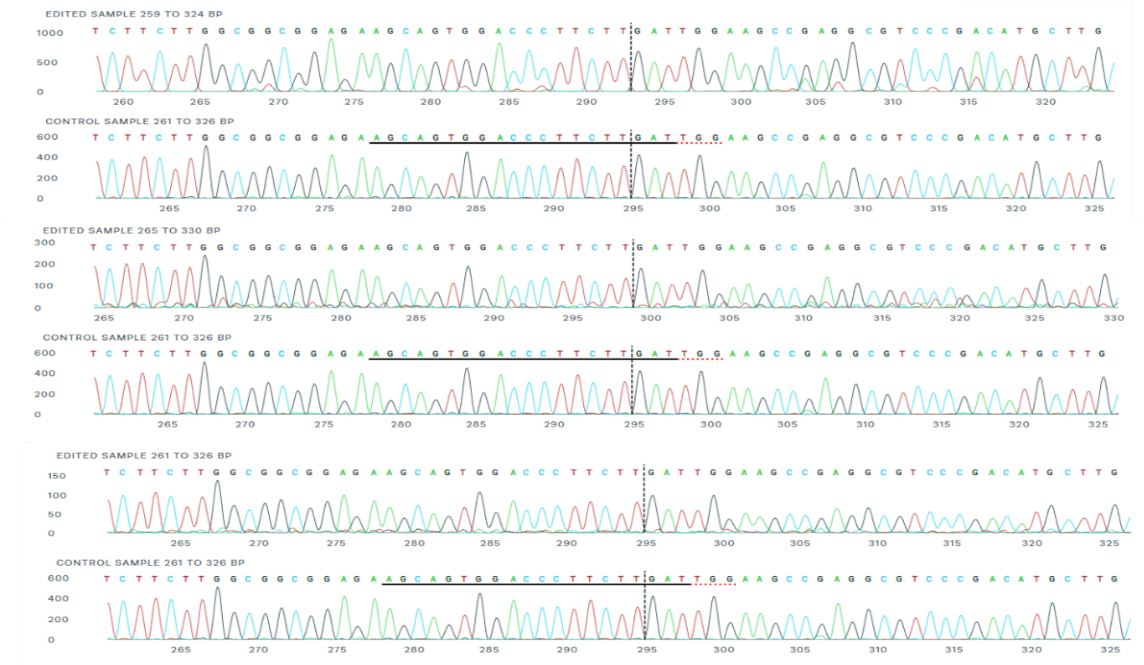
**

BC1

BC8

BC14


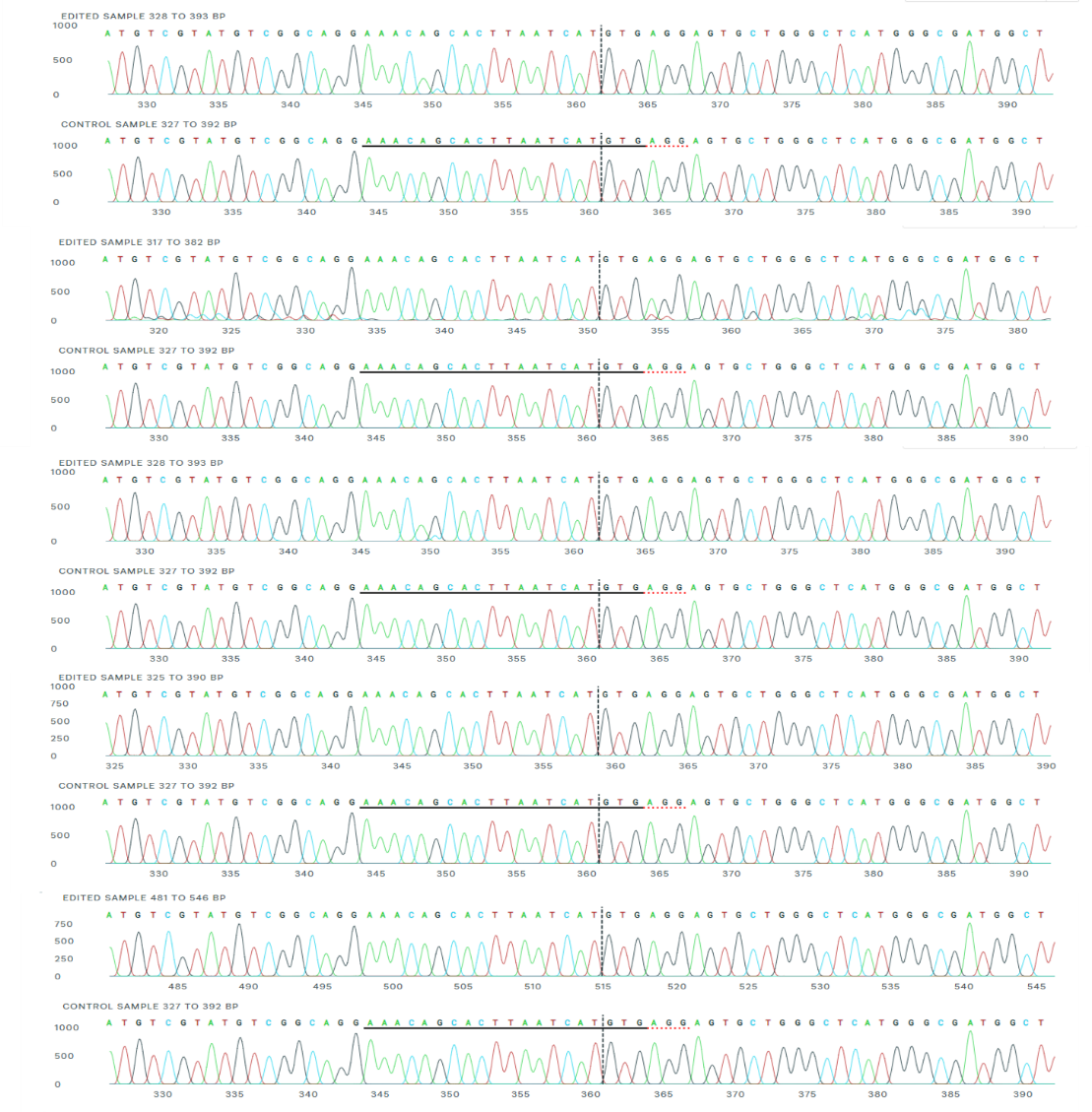


AC18

AC2

AC5

AC6

AC7

**Fig. S5.** ICE traces tab of Cas9-edited genomic DNA containing EgPAT sgRNAs and a control trace for lines derived from bombarded (BC1, BC8 and BC14) and *Agrobacterium* mediated transformation (AC18, AC2, AC5, AC6 and AC7). Cas9 target cut sites are represented by black vertical dashed lines; the guide sequence is underlined; and the sequence of the protospacer adjacent motif (PAM) is marked with a dotted red line.

**Table S1.** sgRNA selection of *EgFAD2* and *EgPAT* genes

| **No** | **sgRNA** | **Target site sequence** | **GC content** | **Secondary Structure** | |
| --- | --- | --- | --- | --- | --- |
|  |  |  |  | **TBP (<12)** | **CBP (<7)** |
| 1 | EgFAD2-T1 | CAGGTACATCAACAACCCCC**CGG** | 60% | 3 | 3 |
| 2 | EgFAD2-T2 | GTACCTGCAGCACACCCACG**CGG** | 65% | 3, 2, 4 | 3, 2, 4 |
| 3 | EgPAT-T1 | AGCAGTGGACCCTTCTTGAT**TGG** | 50% | 5 | 9 |
| 4 | EgPAT-T2 | AAACAGCACTTAATCATGTG**AGG** | 35% | 3, 3, 3, 3 | 3, 7, 3, 9 |

**Table S2.** Primers used in this study

| **No** | **Primer Name** | **Sequence (5'->3’)** | **Purpose** |
| --- | --- | --- | --- |
| 1 | EgPAT#-192F | AAATAGATGGAGGGGTGGACCG | Amplification of *EgPAT* gene target 1 fragments |
| 2 | EgPAT#712R | AGAACCCGCATTTTGGTGAC |  |
| 3 | EgPAT#314F | TAACGACCATCTTCTTGGCGG | Amplification of *EgPAT* gene target 2 fragments |
| 4 | EgPAT#1291R | AGCCTCAACGAAACAAAACATCC |  |
| 5 | EgPAT-F | CCCCACCCCATCATCTTCCT | Amplification and sequencing of *EgPAT* gene fragment |
| 6 | EgPAT-R | CACAGGTTGAGATGCAGGGC |  |
| 7 | EgPAT_S-F | GCGTCGAAGACCATCGGT | Sequencing of WT and mutant of *EgPAT* gene |
| 8 | EgPAT_S-R | GCATGCTTTGTTGTGTAG |  |
| 9 | EgFAD2#28F | AAAGAGCGGGAGGACCAGT | Amplification of *EgFAD2* gene target 1 fragments |
| 10 | EgFAD2#948R | GAACACCCGGTTGAGGACC |  |
| 11 | EgFAD2#275F | CCGCCTGGCCTCTCTACTG | Amplification of *EgFAD2* gene target 2 fragments |
| 12 | EgFAD2#1260R | AAGAGAACACTCACTGGGGGC |  |
| 13 | EgFAD2-F | TTCTCCGACTCCTCCCTCCT | Amplification and sequencing of *EgFAD2* gene fragment |
| 14 | EgFAD2-R | GGGAGAGAAGAAGGGCGTCT |  |
| 15 | EgFAD2-R2 | CCACTAAAATCCCACCCCGC |  |
| 16 | EgFAD2_S-F | CCCTACTTCTCCTGGAAG | Sequencing of WT and mutant of *EgFAD2* gene |
| 17 | EgFAD2_S-R | ATTGTGGAGGGAGGCGAG |  |
| 18 | HYGSCRE-F | ATCGGCACTTTGCATCGG | Amplification of hygromycin selectable marker gene fragment |
| 19 | HYGSCRE-R | CAACCACGGCCTCCAGAAGA |  |
| 20 | Cas9RT-F | CGCTCAGATTGGAGATCAGT | Amplification of *Cas9* gene and RT-PCR of *Cas9p* for expression analysis |
| 21 | Cas9RT-R | CCTGGTGGTGCTCGTCGTAG |  |
| 22 | Cas9P-F | CCTTACTACGTTGGTCCTCTT |  |
| 23 | Cas9P-R | TCTCCTCGATCATCTCCCTATC |  |
| 24 | EgACT2-F | TGCCTGATGGGCAGGTCATCAC | RT-PCR of internal control *EgActin1.* |
| 25 | EgACT2-R | TTCCGGTGCACGATTGCAGGAC |  |
| 26 | SP-L1 | GCGGTGTCATCTATGTTACTAG | Amplification of linked sgRNA expression cassettes and sequence CRISPR plasmids |
| 27 | SP-L2 | GTCGTGCTCCACATGTTGACCG |  |
| 28 | SP-R | GCTGTATCTACGTTATTGAAGC |  |
| 29 | EgFAD2T1Forward | CCTCTAATACGACTCACTATAGGCAGGTACATCAACAACCCCCGTTTAAGAGCTATGC | Forward primer containing the T7 promoter, and a guide sequence used to synthesize the sgRNA template from binary vector by PCR for *in vitro* RNA transcription to produce the sgRNA for cleaving plasmid. |
| 30 | EgFAD2T2Forward | CCTCTAATACGACTCACTATAGGTACCTGCAGCACACCCACGGTTTAAGAGCTATGC |  |
| 31 | EgPATT1Forward | CCTCTAATACGACTCACTATAGGAGCAGTGGACCCTTCTTGATGTTTAAGAGCTATGC |  |
| 32 | EgPATT2Forward | CCTCTAATACGACTCACTATAGGAAACAGCACTTAATCATGTGGTTTAAGAGCTATGC |  |
| 33 | EgFAD2U6aT1F | gccgCAGGTACATCAACAACCCCC | Cloning the guide sequence into the binary vector. The sequences in lower case are the overhangs complementary to  the overhangs of the cloning vector digested by *Bsa*I. |
| 34 | EgFAD2U6aT1R | aaacGGGGGTTGTTGATGCACCTG |  |
| 35 | EgFAD2U6bT2F | gttgTACCTGCAGCACACCCACG |  |
| 36 | EgFAD2U6bT2R | aaacCGTGGGTGTGCTGCAGGTA |  |
| 37 | EgPATU6aT1F | gccgAGCAGTGGACCCTTCTTGAT |  |
| 38 | EgPATU6aT1R | aaacATCAAGAAGGGTCCACTGCT |  |
| 39 | EgPATU6bT2F | gttgAAACAGCACTTAATCATGTG |  |
| 40 | EgPATU6bT2R | aaacCACATGATTAAGTGCTGTTT |  |
| 41 | U-F | CTCCGTTTTACCTGTGGAATCG | Universal primer for amplification of the sgRNA constructs for Gibson Assembly. |
| 42 | gR-R | CGGAGGAAAATTCCATCCAC |  |
| 43 | U-GAL | ACCGGTAAGGCGCGCCGTAGTGCTCGACTAGTATGGAATCGGCAGCAAAGG |  |
| 44 | Pgs-GA2 | CAGGGAGCGGATAACAATTTCACACAGGCACATCCACTCCAAGCTCTTG |  |
| 45 | U-GA2 | GTGCCTGTGTGAAATTGTTATCCGCTCCCTGGAATCGGCAGCAAAGG |  |
| 46 | Pgs-GA3 | CCACGCATACGATTTAGGTGACACTATAGCGCATCCACTCCAAGCTCTTG |  |
| 47 | U-GA3 | CGCTATAGTGTCACCTAAATCGTATGCGTGGTGGAATCGGCAGCAAAGG |  |
| 48 | Pgs-GA4 | GTCGCTAGTTATTGCTCAGCGGCCAAGCTCATCCACTCCAAGCTCTTG |  |
| 49 | U-GA4 | GAGCTTGGCCGCTGAGCAATAACTAGCGACTGGAATCGGCAGCAAAGG |  |
| 50 | Pgs-GAR | TAGCTCGAGAGGCGCGCCAATGATACCGACGCGTATCCATCCACTCCAAGCTCTTG |  |
